# Recent advances in dual response molecular probes for biothiols

**DOI:** 10.1002/smo.20240044

**Published:** 2024-10-21

**Authors:** Master Mwafulirwa, Khamis Abdalla, Wenhai Bian, Hongbei Wei, Liren Xu, Wanyi Yu, Zhang Hui, Qi Yang, Xiaolong Sun

**Affiliations:** ^1^ The Key Laboratory of Biomedical Information Engineering of Ministry of Education School of Life Science and Technology Xi'an Jiaotong University Xi'an China; ^2^ Department of Clinical Engineering Ministry of Health Mzuzu Central Hospital Mzuzu Malawi; ^3^ Ministry of Education and Vocational Training Zanzibar Tanzania

**Keywords:** biothiols, discriminative response, dual‐mode imaging, fluorescent probe, fluorogenic detection

## Abstract

The detection of biothiols such as cysteine (Cys), homocysteine (Hcy), and glutathione (GSH) are critical for understanding their roles in biology and their involvement in various physiological and pathological processes. Recently, significant progress has been made in constructing fluorescent probes capable of detecting and visualizing biothiols. This review provides an in‐depth look at the latest advancements in simultaneous and selective molecular probes, focusing on developments over the last 5 years. We examine design techniques, sensing mechanisms, and imaging methods to assess their effectiveness and responsiveness to thiols. Additionally, we discuss the prevailing challenges and offer recommendations to address them.

## Graphical abstract

This review reports the latest advancements in the development of molecular probes for simultaneous and selective detection of biothiols and bioimaging. The prevailing challenges and recommendations to address them are also discussed.

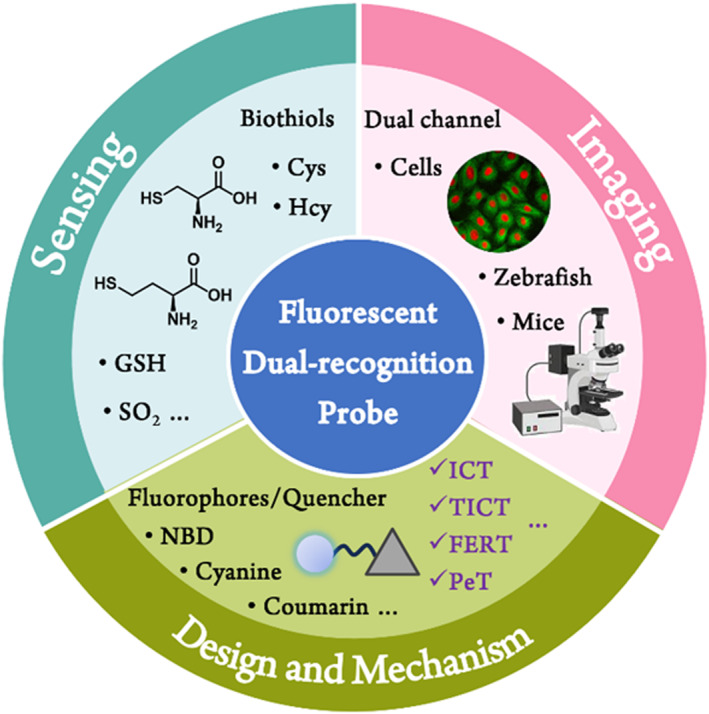

## 1 INTRODUCTION

Biothiols, including cysteine (Cys), homocysteine (Hcy), and glutathione (GSH), are essential for maintaining intracellular oxidative equilibrium in human physiology and serve as significant biomarkers for disease diagnosis and monitoring.[^1^, ^2^] Cys contributes to protein synthesis and the formation of disulfide bonds which are critical for protein structure and function. It also acts as a precursor for the synthesis of other significant biomolecules such as taurine, coenzyme A, and GSH. Elevated levels of Cys have been associated with numerous ailments, including liver failure, edema, growth retardation, and neurological abnormalities.[^3^, ^4^, ^5^, ^6^, ^7^] Hcy is produced during the process of breaking down the amino acid methionine.^[8]^ Increased concentrations of Hcy in the bloodstream have been linked to a range of health disorders, including cardiovascular diseases, Alzheimer's disease, osteoporosis, and deficits in folate and vitamin B_12_.[^9^, ^10^, ^11^, ^12^] GSH, a tripeptide consisting of glutamate, cysteine, and glycine, is the most abundant thiol inside cells and plays a vital role in maintaining cellular redox balance and detoxification activities.[^13^, ^14^] It functions as a powerful antioxidant, safeguarding cells from oxidative stress and preserving the structural integrity of cellular elements. Disruptions in GSH equilibrium are associated with aging, neurological disorders, cancer, and impaired immune system function.[^15^, ^16^, ^17^]

Given the significant importance of biothiols, novel and efficient methods for detecting and monitoring their levels in biological systems are urgently needed. Current detection methods, such as mass spectrometry, chromatographic separation, and electrochemical analysis,[^18^, ^19^] are often cumbersome and costly. Small molecular probes offer a more favorable alternative due to their high sensitivity, non‐invasiveness, affordability, and ease of operation, making them suitable for live cell detection.[^1^, ^20^, ^21^, ^22^]

Over the past 3 decades, scientists have developed numerous fluorescent probes capable of detecting biothiols, allowing for the visualization of biothiol concentration fluctuations under oxidative stress in living cells.[^23^, ^24^, ^25^] In the last decade, research efforts have surged to develop fluorescent probes with dual recognition capabilities for biothiols eliciting discriminative responses.[^26^, ^27^, ^28^, ^29^] Dual‐recognition refers to a probe's ability to exhibit two distinct detections simultaneously or interchangeably. It may also describe bifunctional probes with unique spectral response via the same or two channels based on a single host that can independently detect two different or similar analytes. Molecular fluorescent probes possessing dual recognition sites provide enhanced selectivity, sensitivity, and detection capabilities for biothiols. The engineering paradigm of these probes may incorporate two recognition sites which facilitates a greater degree of interaction between the probe and the analyte of interest. This approach helps mitigate the interference from biomarkers with comparable structures or properties, reducing erroneous positive signals and enhancing detection precision.[^30^, ^31^]

**Scheme 1:**
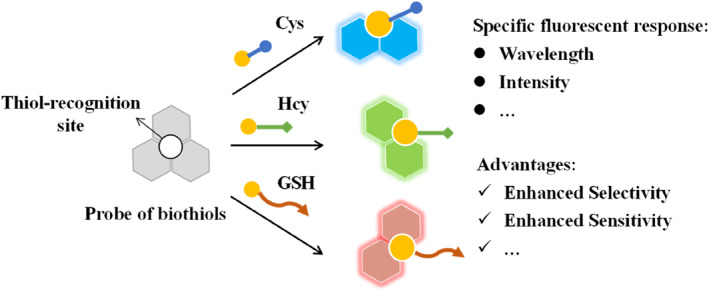
Schematic illustration of the general reaction mechanism of fluorescent probes to discriminate small molecular biothiols.

Numerous scientists have successfully created fluorescent probes with dual detection capabilities, enabling analytes recognition through dual channels or eliciting dual responses.[^27^, ^32^, ^33^, ^34^, ^35^, ^36^, ^37^, ^38^, ^39^, ^40^] Several research groups have reviewed fluorescent probes for detecting different analytes, including biothiols, in single or multi‐channels.[^30^, ^31^, ^41^, ^42^, ^43^, ^44^, ^45^, ^46^, ^47^, ^48^, ^49^] However, our review specifically focuses on molecular probes that can selectively discriminate biothiols by providing dual responses, and imaging biothiols in dual mode (Scheme 1). This review, covering research published in the last 5 years, aims to provide a thorough synopsis of the progress in this exciting field for young researchers.

## 2 DEVELOPMENT AND ADVANCEMENT

Abnormal biothiol levels have been associated with various diseases, such as cardiovascular diseases, hepatic failure, neurodegenerative diseases, cancer, and AIDs, which can contribute to oxidative stress and immune dysfunction.[^50^, ^51^, ^52^, ^53^] Hence, monitoring and accurately determining biothiol levels can provide valuable insights into the pathophysiology, diagnosis, and treatment of these diseases.

In this review, we first classify fluorescent probes that incorporate the nitrobenzoxadiazole group (NBD), and subsequently explore other fluorophores. Further, we evaluate the imaging methods and applications as well as their efficacy and sensitivity in detecting biothiols. Furthermore, we present our analysis of the sector's advancements and pinpoint critical challenges and solutions in this field.

### 2.1 NBD‐based molecular probes

**FIGURE 1:**
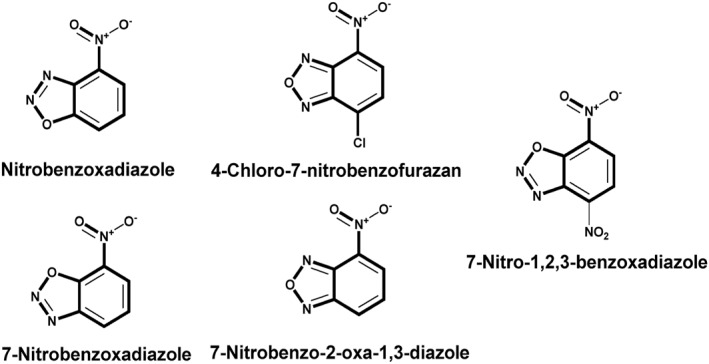
The basic structure of NBD and its derivatives covered in this review.

Several investigations have demonstrated that NBD (Figure 1) is a bio‐analyte sensitive and selective fluorophore.[^54^, ^55^, ^56^, ^57^] The nitro group's remarkable electron‐withdrawing capacity causes a significant red‐shift in its absorption and emission spectra. Because of this characteristic, NBD is especially useful in the NIR range, where biological samples show less auto‐fluorescence and light absorption. When integrated into a probe molecule, NBD can interact or react chemically with target analytes or biomolecules in various ways, modifying NBD's fluorescence characteristics and altering its emission strength, wavelength, or fluorescence lifespan.[^58^, ^59^, ^60^] These properties have led to the development of fluorescent probes that use NBD as a fluorophore moiety to detect biothiols. As a result, we investigated the design and sensing techniques and their sensitivity to thiols. The following is a summary of notable NBD structures and their derivatives for NBD‐based molecular probes.

**FIGURE 2:**
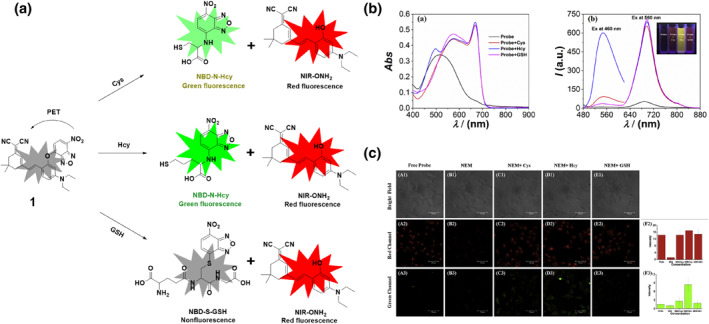
(a) The chemical structure of probe **1** and the sensing mechanism of probe **1** for Hcy compared with Cys and GSH. (b) Absorption and fluorescence spectral changes of probe **1** prior to and after addition of biothiols in DMSO‐PBS. (c) HeLa cells, incubated with probe **1** and pre‐incubated with NEM, exhibited strong emission in both the red and green channels, primarily with Hcy.

In 2019, Xu's group developed probe **1**, a novel hybrid fluorescent probe with dual‐emission responsiveness that combines a dicyanoisophorone‐based fluorophore (NIR‐OH) with 7‐NBD via ether linkage to mask both the NIR fluorophore and the NBD moiety (Figure 2). The sensing mechanism involved a substitution reaction between the probe and biothiols. Interaction with biothiols showed distinct absorption peaks for different thiols, with Hcy inducing a significant 57‐fold fluorescence enhancement, while Cys and GSH exhibited weaker responses. For Hcy, probe **1** caused a notable increase in fluorescence intensity at wavelengths of 549 nm and 697 nm, whereas Cys/GSH mostly led to fluorescence emission at 697 nm. This dual‐emission response enabled the differentiation between Hcy and Cys/GSH. Furthermore, probe **1** exhibited exceptional sensitivity, with detection limits of 33.2 nM for Cys, 33.5 nM for Hcy, and 34.4 nM for GSH. Probe **1** could pass through the cell membrane and offered selective detection and imaging of Hcy and Cys/GSH in living cells.^[61]^

**FIGURE 3:**
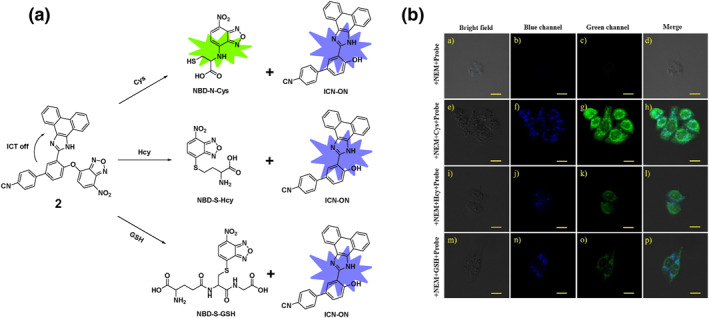
(a) The chemical structure and recognition mechanism of probe **2** towards thiols and (b) confocal fluorescence images of living HeLa cells demonstrate strong fluorescence for Cys in both blue and green channels, effectively differentiating Cys from Hcy/GSH.

Around the same time in 2019, following the development of probe **1**, the Lu group designed and synthesized a dual‐emission turn‐on probe **2** (Figure 3). The probe utilized a simple ether bond linking 7‐nitro‐1,2,3‐benzoxadiazole (NBD) and a cyano‐phenanthroimidazole fluorophore, aiming to detect and distinguish Cys from Hcy/GSH. The probes' ether bond could be readily broken using an aromatic nucleophilic substitution reaction, releasing the fluorophore ICN‐OH and NBD‐containing intermediates, which resulted in blue fluorescence at 470 nm (excited at 365 nm) by activating intramolecular charge transfer (ICT). Upon exposure to Cys, probe **2** exhibited two fluorescence signals at wavelengths of 470 and 550 nm under excitation wavelengths of 365 and 480 nm, respectively. Conversely, the introduction of Hcy and GSH only caused a noticeable increase in blue fluorescence at 470 nm. This differential response, possibly due to geometric limitations or solvent influence leading to the rearrangement of NBD‐S‐Hcy/GSH to NBD‐N‐Hcy/GSH, enabled probe **2** to differentiate Cys from Hcy/GSH in both the blue and green channels. The limits of detection for Cys, Hcy, and GSH were estimated to be 22.6 nM, 31.2 nM, and 17.7 nM, respectively. These results demonstrated that probe **2** could accurately measure biothiols and distinguish between Cys, Hcy, and GSH. Additionally, probe **2** effectively visualized biothiols in both living cells and zebrafish models, showcasing its potential for use in biological applications.^[62]^

**FIGURE 4:**
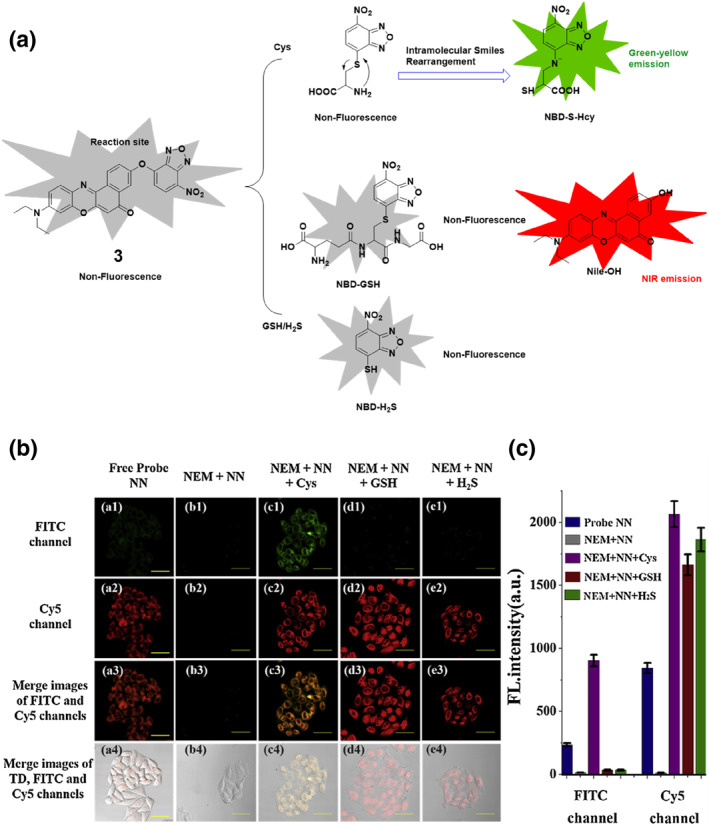
(a) The chemical structure of probe **3** and proposed sensing mechanism of probe **3** to Cys over GSH/H_2_S. (b) In HeLa cells, confocal fluorescence images of probe **3** responding to Cys, GSH, and H_2_S show a significant enhancement of fluorescence in both the FITC and Cy5 channels, distinguishing Cys from GSH/H_2_S. (c) The quantification of fluorescence intensity of the FITC and Cy5 channels of (b).

In terms of reaction mechanism, the interaction between NBD and the probe is critical to understand the design and functionality of these fluorescent probes. Subsequently, the Zhu group developed a NIR fluorescent probe **3** with the ability to differentiate Cys from GSH and hydrogen sulfide (H_2_S) both in vivo and in vitro (Figure 4). This probe harnessed Nile‐OH and NBD synergistically as the fluorescent reporter group and recognition site, respectively. Initially, probe **3** exhibited no fluorescence within the visible‐to‐NIR range. However, upon encountering Cys, it exhibited a substantial enhancement in fluorescence in both the green and NIR emission spectra. In contrast, exposure to GSH or H_2_S only resulted in an increase in fluorescence in the NIR range. The cell imaging results with probe **3** demonstrated its efficacy in distinguishing Cys from GSH/H_2_S in HeLa cells. Pre‐treatment with *N*‐ethylmaleimide (NEM) and probe **3** followed by Cys exposure led to significant fluorescence enhancement in both fluorescein isothiocyanate (FITC) and Cyanine 5 (Cy5) channels, while GSH/H_2_S treatment resulted in strong fluorescence mainly in the Cy5 channel, showcasing the probe's capability for selective biothiol differentiation within cellular environments. This dual‐channel responsive mode allowed for the accurate differentiation of Cys from GSH and H_2_S. Furthermore, probe **3** demonstrated high selectivity, stability, and low limits of detection of 0.05 μM, 0.11 μM, and 0.02 μM for Cys, GSH, and H_2_S, respectively. Significantly, this investigation enabled the first‐ever in vivo differentiation of Cys from GSH/H_2_S.^[63]^

**FIGURE 5:**
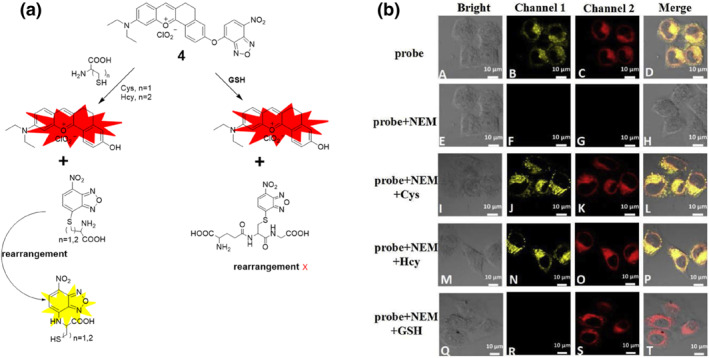
(a) The chemical structure of probe **4** and the response mechanism of probe **4** with biothiols. (b) Cell images of Cys, Hcy, and GSH with probe **4** have the ability to detect exogenous Cys/Hcy and GSH from the dual emission channel in cells.

Commencing with insights into the sensing and design aspects of NBD and the probe, the Huang group developed a red fluorescent probe **4** with dual channels to detect the presence of Cys/Hcy and GSH in plants (Figure 5). This probe was created by connecting NBD‐Cl with a near‐red dye (compound 1) via an ether bond which serves as one of the probable sites for the thiol‐promoting dual reaction. The sensing mechanism involved nucleophilic substitution between biothiols and probe **4**. When Cys/Hcy reacted with probe **4,** a nucleophilic substitution process occurred, resulting in the liberation of fluorescent compound and the generation of a unique fluorescence emission at 625 nm. In contrast, GSH could not cause intramolecular rearrangement in the NBD‐thiol adduct due to steric hindrance, leading to a distinct fluorescence reaction. As a result, the probe displayed two different fluorescence emissions, enabling the detection of various thiols by analyzing their unique fluorescence signals. The dual‐channel fluorescence emission allowed for the specific detection and quantification of Cys, Hcy, and GSH in zebrafish and cells in the analyzed samples. Furthermore, probe **4**'s unique fluorescence signal made it suitable for identifying Cys/Hcy and GSH in *Arabidopsis thaliana*, a model plant species.^[64]^

**FIGURE 6:**
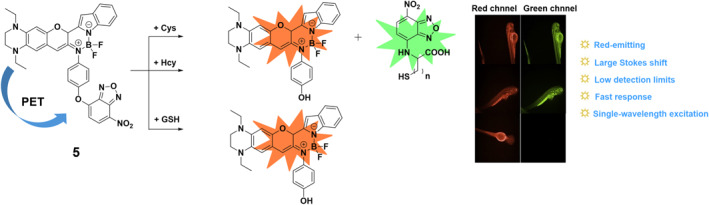
The chemical structure of probe **5** and the sensing mechanism and response process of probe **5** towards zebrafish. Probe **5** was constructed for simultaneously distinguishing Cys/Hcy and GSH under a single‐wavelength excitation and displayed distinct fluorescence toward Cys/Hcy and GSH.

Towards the end of 2019, the Ren group synthesized a red‐emitting fluorescent probe **5** designed to discriminate between Cys/Hcy and GSH (Figure 6). Probe **5** was based on an iminocoumarin borate complex and exhibited a large Stokes shift of 100 nm. The probe responded to Cys/Hcy by displaying two well‐separated emission bands at 565 and 630 nm, while it observed a single emission band at 630 nm for GSH. Selective detection was achieved by incorporating a thiol sensing group, NBD. The THQ donor and the NBD acceptor efficiently transferred charge, making the fluorescence signals for the various thiols distinct. Probe **5** exhibited high sensitivity with detection limits below 0.095 μM and rapid response times of less than 8 min. Moreover, the probe demonstrated the ability to differentiate between Cys/Hcy and GSH in both living cells and zebrafish.^[65]^

**FIGURE 7:**
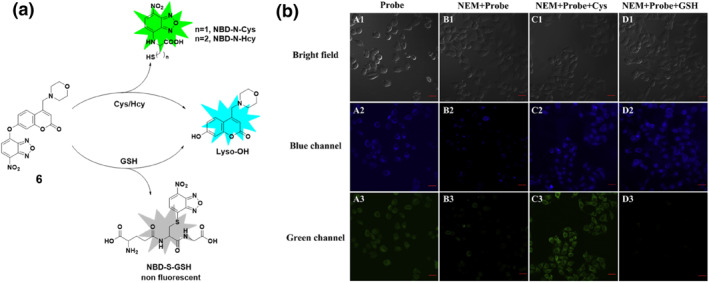
(a) The chemical structure of probe **6** and the reaction mechanism of probe **6** for biothiols by dual channels. (b) Confocal laser scanning microscope images of HeLa cells demonstrated that probe **6** effectively detected Cys and GSH within living cells using dual fluorescence channels. Both the blue and green channels displayed robust fluorescence signals specifically for Cys.

In the realm of sensing mechanisms, characterized by the strategic integration of NBD derivatives and a fluorophore within the probe, the Song group unveiled probe **6** in early 2020 (Figure 7). The design involved using coumarin as a fluorophore and NBD derivatives as a recognition unit with a morpholine moiety incorporated for lysosomal targeting. The sensing mechanism relied on the nucleophilic substitution‐rearrangement reaction of NBD derivatives with Cys/Hcy which distinguished them from GSH. Probe **6** demonstrated exceptional selectivity and sensitivity towards Cys/Hcy and GSH compared to other amino acids and ions when stimulated at two distinct wavelengths (400 and 470 nm). In dual‐channel detection, the probe displayed high selectivity and sensitivity, enabling imaging of biothiols in lysosomes. Furthermore, probe **6** could be used for simultaneous detection and discrimination of Cys/Hcy and GSH in the lysosomes of living cells with low cytotoxicity.^[2]^

**FIGURE 8:**
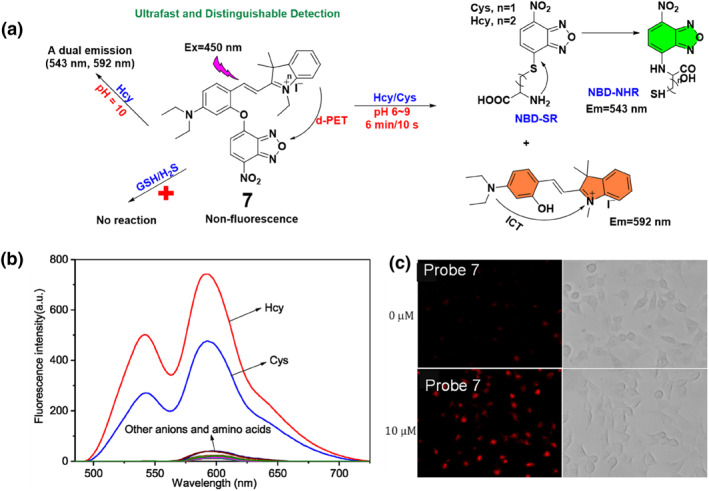
The chemical structure and proposed response mechanism of probe **7** for Hcy/Cys (a) Fluorescence intensity of probe **7** upon addition of Hcy, Cys, amino acids and other competing anions (b) and Imaging probe **7** targeting Hcy within living cells demonstrates its ability to penetrate the cell membrane and monitor thiol levels effectively (c).

Utilizing NBD as a pivotal reactive site within the probe and harnessing advanced sensing mechanisms, the Zhong group developed a novel fluorescent probe **7** using a D‐A (donor‐acceptor) system to specifically recognize biothiols (Figure 8). The sensing mechanism involved the cleavage of the ether bond in the NBD unit by the thiol group of Hcy/Cys through an aromatic nucleophilic substitution reaction, resulting in the release of molecule 1 and NBD‐SR. The design principle focused on utilizing pH and reaction time as factors to differentiate between Hcy and Cys enabling their individual detection. Probe **7** had the capability to detect Hcy and Cys across a pH range of 6–9, with extremely low detection limits of 2.81 and 2.33 μM, respectively. The fluorescence spectra of probe **7** revealed its selectivity towards Hcy and Cys. While probe **7** initially showed minimal fluorescence due to a donor‐excited photoinduced electron transfer process, significant fluorescence enhancements at 543 and 592 nm were observed upon treatment with Hcy/Cys. This distinct fluorescence response highlights the specific recognition capability of probe **7** towards Hcy and Cys compared to other tested anions and amino acids. Additionally, probe **7** exhibited high selectivity for Cys, allowing for rapid detection within a time frame of 10 s, whereas Hcy detection required a longer period of around 6 min. This difference in response time facilitated the discernible identification of Hcy and Cys. They effectively used probe **7** for biological imaging, specifically to detect and visualize Hcy in living cells.^[66]^

**FIGURE 9:**
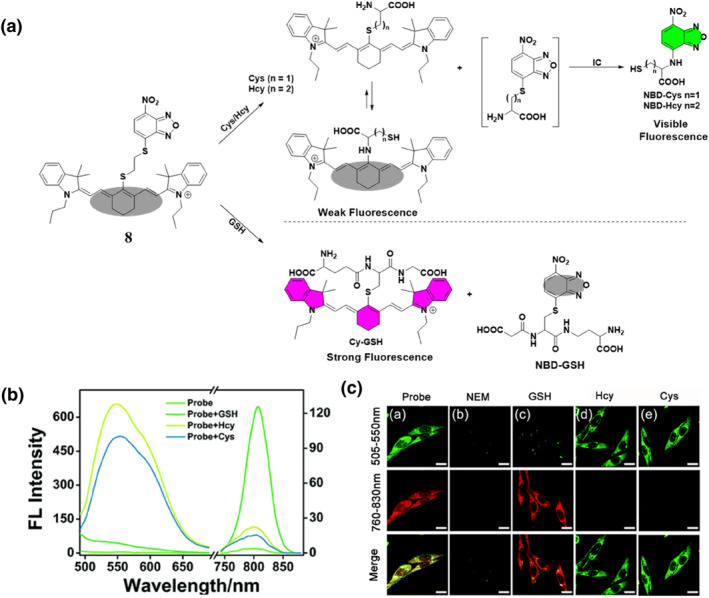
The response mechanism of probe **8** for discriminating between biothiols with two distinct emission patterns (top). Spectral properties of probe **8** to thiols (b). Visualization of biothiols in U87 cells to differentiate between Cys/Hcy and GSH via dual emission channels in living cells (c).

Drawing on advancements in NBD and molecular probes, the Li group made significant strides in 2020 by pioneering an innovative fluorescent probe **8** tailored for the precise detection of biothiols with remarkable sensitivity and precision (Figure 9). They introduced two enhanced fluorescent probes (Cy1 and Cy2), both of which exhibited improved sensitivity and the ability to distinguish between Cys/Hcy and GSH through dual emission activation. The probes were constructed using IR‐780 and NBD, connected by an ether bond. The Cy2 probe responded to GSH with near‐infrared (NIR) emission at 810 nm (λ_ex_ = 720 nm) and to Cys/Hcy with visible green emission at 550 nm (λ_ex_ = 470 nm). In the absence of biothiols, Cy2 exhibited minimal fluorescence due to an efficient PET process induced by the NBD moiety (Figure 9b). Upon the addition of Cys or Hcy, a blue‐shifted peak at 460 nm appeared, accompanied by emission bands at 550 nm, resembling reported NBD dyes. Notably, the introduction of GSH resulted in a significant red‐shift in the fluorescence peak (λ_em_: 750–850 nm) with a remarkable fluorescence enhancement of approximately 45‐fold, demonstrating Cy2's strong selectivity towards thiols and its ability to distinguish between Cys/Hcy and GSH effectively. When tested with human serum samples, the Cy2 probe demonstrated its functionality, indicating potential diagnostic applications. Furthermore, probe **8** showed the ability to visualize both exogenous and endogenous biothiols in living cells using fluorescence microscopy. The research team conducted bioimaging investigations with probe **8** in mice with tumors and mouse models with acute liver injury, yielding promising results. This study highlighted the practicality of probe **8** as a valuable tool for bioimaging.^[67]^

**FIGURE 10:**
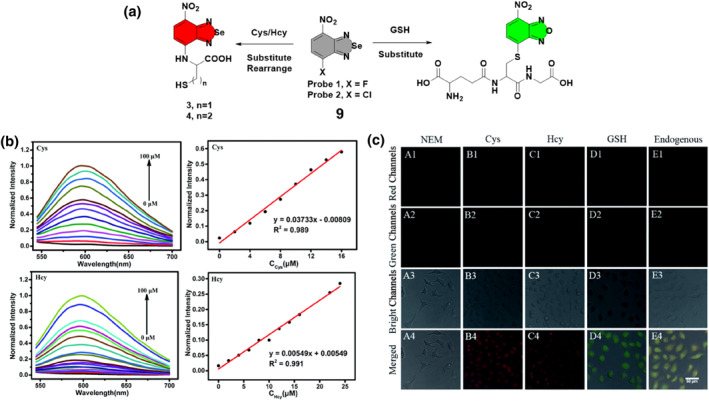
The response mechanisms of probe **9** for distinguishing the detection of biothiols. Fluorescence intensity spectra (left) of probe **9** in the presence of thiols and the linear changes of the fluorescence intensity (right) of probe **9** at 600 nm and as a function of Cys and Hcy concentration (b). Confocal fluorescence images of endogenous and exogenous thiols in HepG2 cells (c).

Breaking new ground in fluorescent probe development, the Yao group developed a small fluorescent probe **9** that could detect Cys, Hcy, and GSH concurrently in 2021 (Figure 10). The investigation employed a single‐atom substitution approach, inserting a selenium atom into a 4‐chloro‐substituted NBD (NBD‐Cl) fluorophore. This substitution enabled the probe to differentiate Cys/Hcy from GSH, allowing for the visualization of Cys/Hcy and GSH in live cells using red and green emission channels, respectively. The detection process involved replacing chloride with thiolate in the presence of Cys/Hcy, resulting in the creation of amino‐substituted products that displayed intense fluorescence. The fluorescence emission band at 600 nm increased progressively with increasing amounts of Cys/Hcy when excited at 510 nm, distinguishing probe **9** from other reported probes. Probe **9** exhibited strong linearity with Cys (0–16 μM) and Hcy (0–25 μM) concentrations, with correlation coefficients of 0.989 and 0.991, respectively, and showed a linear relationship with GSH (0–8 μM) with a correlation coefficient of 0.992. The limits of detection were 249.9 nM for Cys, 1.314 μM for Hcy, and 19.5 nM for GSH, indicating probe **9**'s efficacy in detecting Cys/Hcy and GSH with high sensitivity and accuracy. Nevertheless, GSH, which forms a sulfur‐substituted compound, did not display fluorescence. The introduction of a selenium atom enhanced the spectral characteristics of probe **9**, leading to improved fluorescence emission and was further applied in fluorescence imaging of biothiols in living cells.^[68]^

**FIGURE 11:**
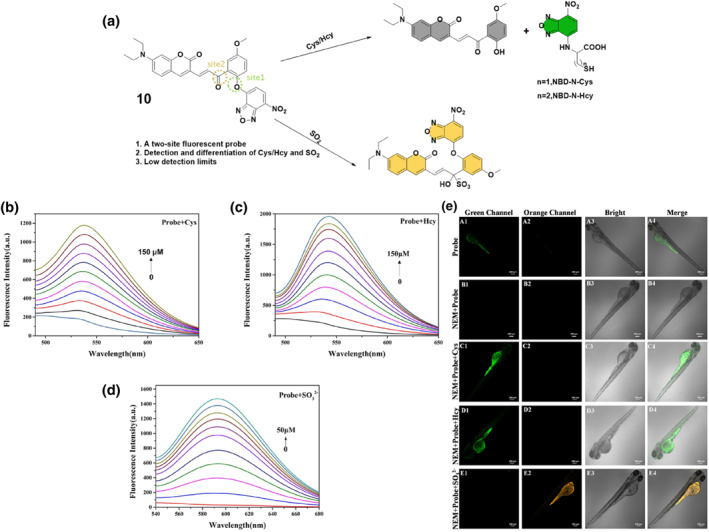
Dual site probe **10** for detecting and discriminating biothiols (top). Fluorescence titration of probe **10** in DMSO/PBS (b, c, d). Imaging of probe **10** in zebrafish to detect and distinguish Cys/Hcy and SO_2_ in vivo via the green and orange channels (e).

A year later in 2022, the Zhang group designed probe **10** for the detection of Cys, Hcy, and SO_2_. This probe consisted of a coumarin derivative and an NBD moiety, which underwent specific reactions with Cys/Hcy and SO_2_ (Figure 11). The reaction mechanism involved the cleavage of the ether bond in probe **10** by Cys/Hcy, resulting in the emission of green fluorescence at a wavelength of 540 nm. Conversely, the reaction between SO_2_ and probe **10** released orange fluorescence at a wavelength of 590 nm. The probe's design principle relied on the distinct reaction sites for Cys/Hcy and SO_2_, enabling their differentiation and selective detection. The spectral analysis of probe **10** revealed distinct responses to Cys/Hcy and SO_3_^2−^. Absorption peaks shifted and new peaks emerged upon the addition of Cys/Hcy and SO_3_^2−^, leading to enhanced fluorescence at specific wavelengths. Probe **10** displayed low fluorescence quantum yield initially but showed improved sensitivity and selectivity towards Cys, Hcy, and SO_3_^2−^, with detection limits of 0.25 μM for Cys, 0.14 μM for Hcy, and 0.05 μM for SO_3_^2−^. The probe was effectively used to visualize Cys/Hcy and SO_2_ in HepG‐2 cells and zebrafish.^[69]^

**FIGURE 12:**
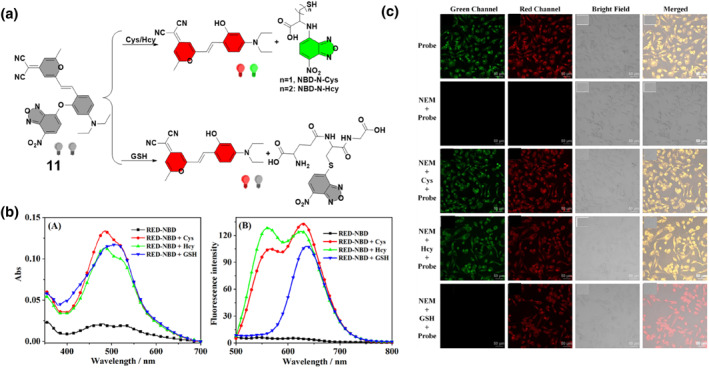
Dual‐emission fluorescent probe **11** for discriminative sensing of biothiols and cell imaging. Absorption (a) and emission spectra (b) of probe **11** in the absence and the presence of thiols. Fluorescent images of Hela cells differentiating Cys/Hcy and GSH in the green and red channels (c).

In a notable advancement in dual‐response molecular probe research, probe **11** was developed by the Hao group through the conjugation of the red‐emitting fluorophore RED with NBD in 2022. Probe **11** exhibited distinctive spectral responses to biothiols, with strong absorption peaks at 480 and 520 nm observed after interactions, indicating the formation of NBD‐biothiol and RED products (Figure 12). While probe **11** initially showed weak fluorescence, it displayed significant fluorescence enhancement in both green and red channels upon reacting with Cys/Hcy and solely in the red channel with GSH, enabling the differential detection of Cys/Hcy and GSH. The results suggest probe **11**'s potential for selective biothiol detection through dual‐emission fluorescence analysis, requiring only a single excitation source. Upon reaction with Cys/Hcy, probe **11** cleaved into RED and NBD‐Cys/Hcy, exhibiting distinct red and green fluorescence. In contrast, the reaction with GSH generated non‐fluorescent NBD‐SH. The design principle involved combining NBD's reactivity with the desirable optical properties of RED, enabling discriminative sensing of biothiols. Probe **11** demonstrated high sensitivity and low detection limits, effectively distinguishing between Cys/Hcy and GSH. Additionally, the group employed probe **11** to visualize Cys/Hcy and GSH within cells.^[70]^

**FIGURE 13:**
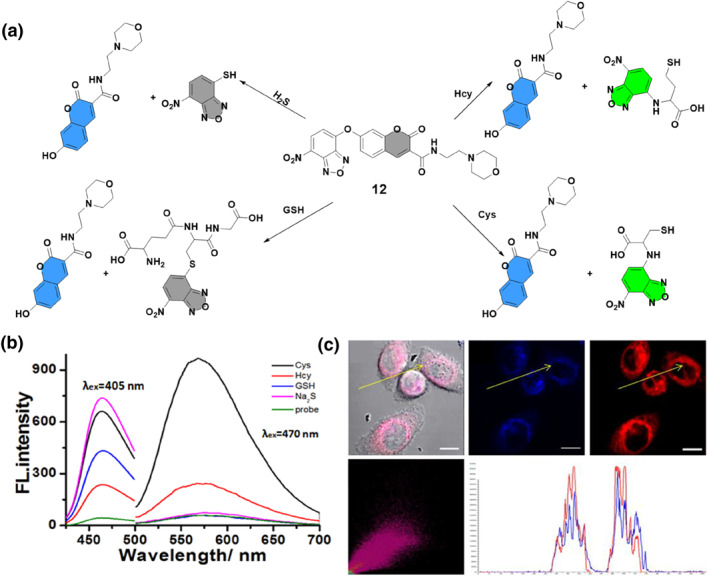
A response mechanism of probe **12** to thiols (a). The emission spectra of probe **12** (b) with or without analytes in PBS solution (b). Imaging of live cells with probe **12** and Lyso‐Tracker Deep Red (c).

Lysosomes are cellular structures that break down both intracellular and extracellular substances using diverse molecular mechanisms.^[58]^ They also repair damaged substances and provide intermediate compounds for additional metabolic processes. Lysosomes contain about 50 types of enzymes that facilitate breakdown, with an optimal pH range of 4.5–5.5, which sustains enzyme activity. In lysosomes, biothiols play a crucial role in proteolysis, particularly in breaking down disulfide bonds. For instance, the stability of the lysosomal membrane may be associated with the role of GSH and Cys as stimulators for the degradation of albumin in liver lysosomes. Hence, identifying and distinguishing biothiols in lysosomes holds great significance for understanding their physiological functions and precisely diagnosing lysosomal‐associated disorders in living organisms.[^71^, ^72^, ^73^, ^74^, ^75^, ^76^] In this study, the Jing group constructed a lysosome‐targeted dual‐emission fluorescence probe **12** that incorporated coumarin, NBD, and morpholine groups (Figure 13).^[58]^ Upon exposure to biothiols, the ether bond underwent cleavage, resulting in the release of the coumarin fluorophore, which emitted blue fluorescence at 465 nm when excited at 405 nm. The NBD chromophore, specifically NBD‐Cys, exhibited intense green fluorescence. In contrast, NBD‐Hcy displayed weak green fluorescence, while NBD‐GSH/NBD‐SH did not exhibit any green signal at 565 nm when excited at 470 nm. This dual‐color mode enabled the probe to differentiate between Cys and Hcy/GSH, as well as H_2_S, in a PBS solution. Furthermore, probe **12** was employed to visualize exogenous biothiols in HeLa cells and successfully distinguished Cys from Hcy/GSH in a zebrafish model that also involved H_2_S.

**FIGURE 14:**
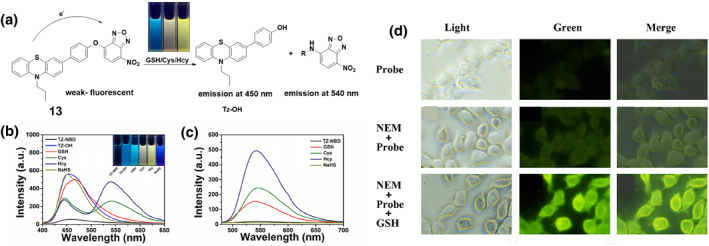
(a) Probe **13** for the detection of thiols (top). (b) Fluorescence spectra (λ_ex_ = 320 nm) of probe **13** with and without biothiols (c) Fluorescence spectra (λ_ex_ = 450 nm) of probe **13** with and without biothiols. (d) Fluorescence images of HepG2 cells in the green channel.

A year later in 2023, the Tu group developed probe **13** specifically to detect biothiols, with a focus on GSH (Figure 14). The probe employed phenothiazine and 7‐NBD fluorophores, and its reaction mechanism involved the occurrence of PET between phenothiazine and 7‐NBD. In the absence of biothiols, the probe did not emit fluorescence. However, when biothiols were present, probe **13** experienced structural alterations, leading to the creation of unique fluorescence hues. The photophysical properties of probe **13** in the presence of sulfur species like GSH, Cys, Hcy, and NaHS were investigated. Upon interaction with these species, distinct changes in absorption spectra were observed, with new absorption bands appearing at varying wavelengths. The fluorescence spectra showed significant emission peaks at 450 and 540 nm upon addition of biothiols, with discernible differences in emission characteristics for GSH, Cys, and Hcy, indicating the probe's ability to selectively detect these active thiols based on their unique fluorescence responses. The fluorescence activation mechanism of probe **13** involved intramolecular cyclization induced by Cys or Hcy, leading to enhanced fluorescence, while GSH and NaHS induced different fluorescence patterns, enabling the differentiation of these sulfur species based on their fluorescence signatures. The probe demonstrated exceptional specificity and sensitivity towards thiols, with a remarkably low detection limit of 0.207 μM for GSH. Crucially, HepG2 cells effectively utilized probe **13** for GSH imaging, highlighting its potential as a highly promising fluorescent probe for the precise identification and visualization of biothiols.^[77]^

**FIGURE 15:**
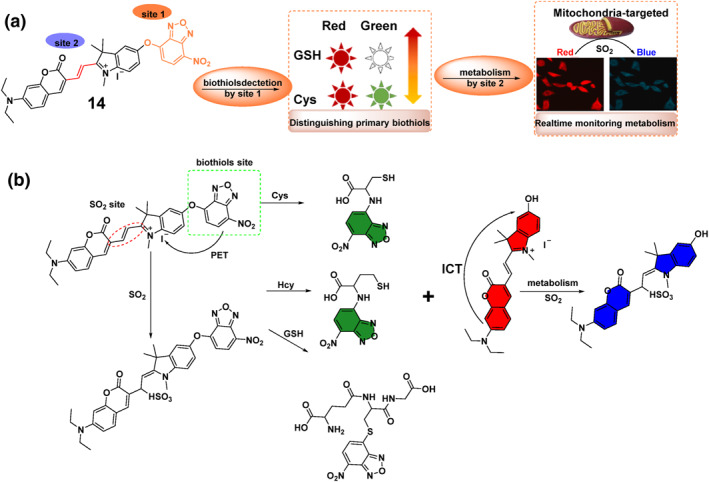
A new strategy of probe **14** for distinguishing biothiols by the ratio fluorescence change of red‐green and real‐time monitoring metabolism by the ratio fluorescence change of red‐blue (a). Response mechanisms of probe **14** with Cys/Hcy, GSH, and SO_2_, with three well‐defined emission bands (b).

In the same year of 2023, the Lan group created a fluorescent probe **14** that could target mitochondria and detect biothiols (Figure 15). This probe allowed for the simultaneous differentiation of biothiols and the visualization of their metabolism in cancer cells and tumor models. Probe **14** was specifically engineered with two recognition sites to identify and differentiate between Cys, Hcy, GSH, and SO_2_ derivatives. Probe **14**, a mitochondria‐targeted NIR fluorescent compound, comprised a coumarin‐hemicyanine fluorophore and an NBD chromophore linked by an ether bond.^[78]^ It utilized an ICT‐PET synergetic mechanism, with dual recognition sites for detecting Cys/Hcy (Red‐Green), GSH (Green), and SO_3_^2−^ (Blue) via three channels. Initially, it differentiated between Cys and GSH by detecting alterations in the ratio of dual defined emission bands (Red‐Green). Then, it utilized ratiometric fluorescence (Red‐Blue) to visualize the metabolism of biothiols by imaging the formation of SO_2_. Probe **14** was effectively utilized to monitor the metabolic process of GSH in MCF‐7 cells and the metabolism of biothiols in breast cancer tumor models, implying that alterations in biothiol metabolism could function as diagnostic markers during cancer therapy.

**FIGURE 16:**
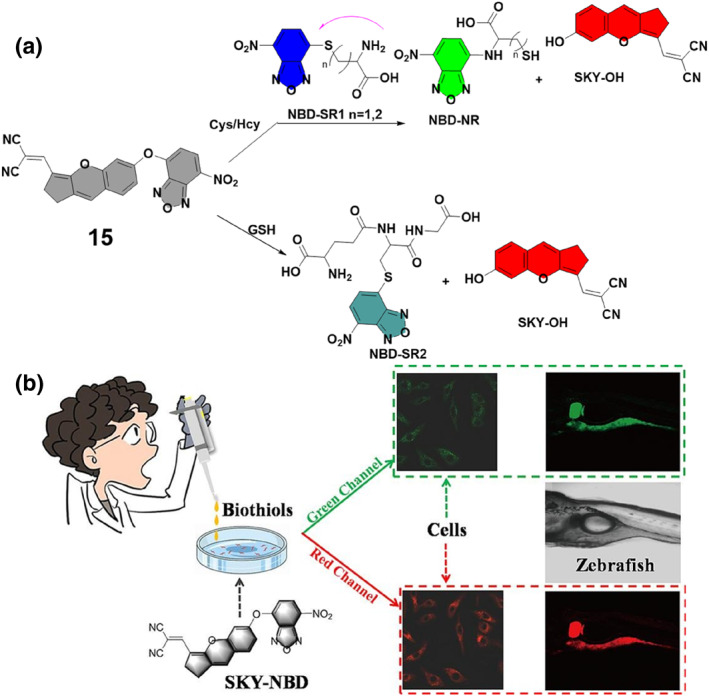
The response mechanism between probe **15** and biothiols (a), along with its application for highly efficient biothiol imaging in living samples (b).

Towards the end of 2023, the Shen group developed a highly sensitive dual‐channel responsive fluorescent probe **15** for the detection of biothiols in both in vitro and in vivo (Figure 16). Probe **15** comprised two fluorophores: NBD‐Cl and SKY‐OH. The reaction mechanism involved combining NBD‐Cl with SKY‐OH to generate probe **15**. The electron‐withdrawing function of the NBD moiety initially inhibited fluorescence. However, when Cys or Hcy were present, the probe reacted with these biothiols, resulting in the liberation of SKY‐OH and NBD‐NR, which led to a substantial increase in fluorescence in both the red and green channels. However, only SKY‐OH was released in the presence of GSH, leading to a significant fluorescence emission in the red channel. Probe **15** effectively visualized biological thiols in living cells and zebrafish larvae, indicating its potential for investigating disorders linked to biothiols in living organisms.^[79]^

### 2.2 Dual‐recognition molecular probes based on other fluorophores

In recent years, the development of fluorescent molecular probes has revolutionized the field of bioimaging and clinical diagnosis. These probes, composed of various fluorophores and functional groups, offer unique capabilities for targeted detection and visualization of biomolecules within living systems. There are clear benefits to fluorescent molecular probes that contain other fluorophores, like naphthalimide, dansyl, cyanine, and dicyanomethylene‐4H‐pyran. For instance, Dicyanomethylene‐4H‐pyran, known for its versatility, provides enhanced fluorescence quantum yield and improved stability.[^80^, ^81^] Cyanine dyes, on the other hand, offer a wide range of emission wavelengths, enabling multiplexed imaging and probe development with superior brightness and photostability^[82]^ while naphthalimide exhibits more intense fluorescence and better photostability.[^83^, ^84^] These fluorophores serve as key components in the design of dual‐recognition probes, facilitating the specific tracking of biothiols in living cells.

**FIGURE 17:**
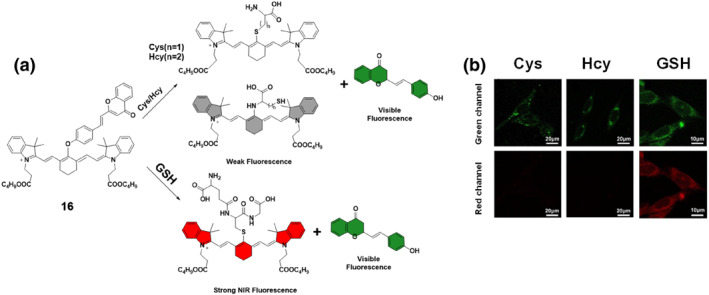
Probe **16**'s reaction with biothiols (a) and its use for highly effective biothiol imaging in green and red channels (b).

Fluorescence reporting units, essential for emitting fluorescence in response to specific triggers, play a crucial role in sensing and imaging applications. Leveraging the distinct properties of dicyanomethylene‐4H‐pyran, renowned for its robust fluorescence and responsiveness to environmental cues, the Xu group engineered a specialized fluorescent probe, probe **16**. The group tailored this probe, featuring dual channels, to precisely monitor GSH within mitochondria by integrating dicyanomethylene‐4H‐pyran and cyanine linked through an ether linker (Figure 17). The sensing mechanism of probe **16** relied on its reactivity towards thiol groups. The probe demonstrated intense fluorescence exclusively upon interaction with GSH. This targeted GSH reaction enabled the tracking and detection of mitochondrial GSH, displaying clear emission bands in both visible and NIR channels. Experimental studies conducted to assess the in vitro specificity of the probe for GSH involved pretreating U87 cells with NEM, a thiol‐blocking reagent. Subsequent incubation with probe **16** revealed no significant green and red fluorescence in NEM‐treated cells, indicating the blockage of GSH detection. When Cys and Hcy were introduced along with GSH, similar fluorescence intensity was observed in the green channel, but distinctive differences were noted in the red channel, where GSH exhibited stronger fluorescence signals. This selective fluorescence response in dual channels allowed for the monitoring of endogenous GSH levels with minimal interference from Cys and Hcy, enhancing the accuracy of GSH detection in cellular environments. Additionally, probe **16** was successfully used to detect solid tumors using both naked‐eye and NIR imaging, showcasing its potential for clinical diagnosis applications.^[85]^

**FIGURE 18:**
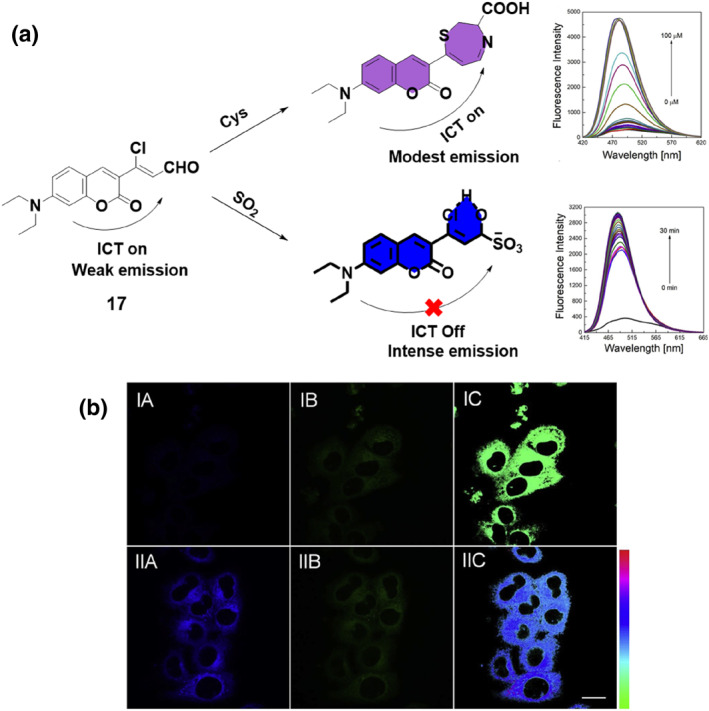
Probe **17**'s design strategy based on dual quenching mechanisms (a). The fluorescence images of HeLa cells pre‐incubated with thiols and stained with probe **17** (b).

The Zhang group developed a ratiometric probe **17** that utilized dual quenching methods to specifically visualize intracellular SO_2_, effectively eliminating interference from Cys (Figure 18). The probe's sensing techniques encompassed ICT and internal rotation of a vinyl group. The investigation demonstrated the specific identification of SO_2_ by obstructing the two simultaneous processes that reduce fluorescence, leading to a highly pronounced shift towards the blue end of the emission spectrum. The fluorescence spectra of 5 μM probe **17** in the presence of varying concentrations of NaHSO_3_ (0–100 μM) for 30 min demonstrated a gradual increase in the fluorescence intensity with increasing SO_2_ concentration, reaching equilibrium around 80–100 μM. This indicated the sensitivity of probe **17** to SO_2_, as the fluorescence response correlated with the concentration of the analyte, showcasing the probe's capability for detecting and quantifying SO_2_ levels with a calculated detection limit of 20 μM, underlining the high selectivity of the probe for SO_2_ detection. The probe demonstrated exceptional selectivity, sensitivity, rapid response, and minimal cytotoxicity, illustrating its successful use for ratiometric imaging of intracellular SO_2_, effectively overcoming interference from Cys.^[86]^

**FIGURE 19:**
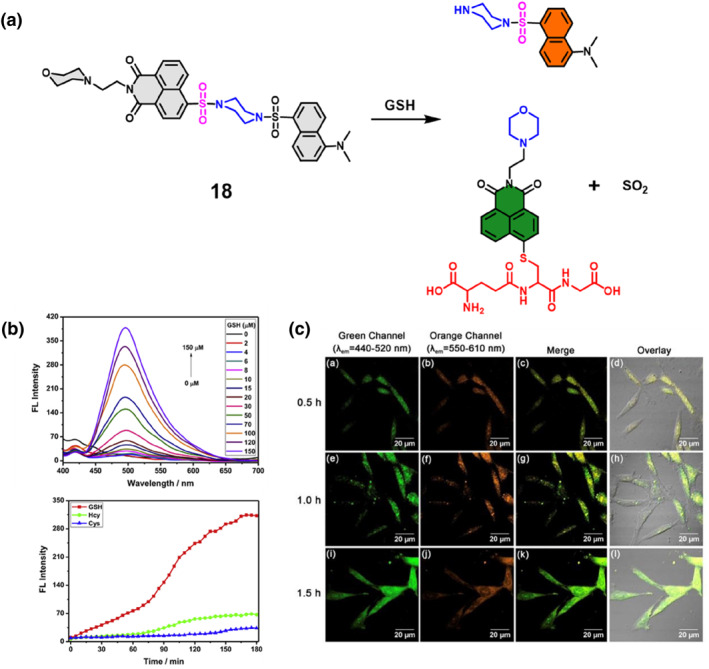
The design principle and proposed reaction mechanism of probe **18** towards GSH (a). The fluorescence titration spectra of probe **18** show changes in intensity over time at 495 nm as GSH concentrations in HEPES buffer are added (b). Probe **18** with the capability to track intracellular thiol in a dual‐emission manner (c).

Naphthalimide‐sulfonamide and dansyl‐sulfonamide, distinguished by their unique fluorescence profiles and sensitivity, are strategically harnessed in fluorescent probes for accurate biomolecule tracking in biological contexts. In 2019, the Xu group developed a fluorescent probe **18** by combining naphthalimide‐sulfonamide and dansyl‐sulfonamide to track GSH in lysosomes (Figure 19a). Probe **18** exhibited dual‐emission properties, where the green emission was associated with the GSH‐linked naphthalimide and the orange emission originated from the cleavage product of dansyl. Researchers created this probe by modifying two fluorophores, naphthalimide and dansyl, to achieve high selectivity towards GSH. The fluorescence titration spectra of 10 μM probe **18** upon the addition of varying concentrations of GSH (0–150 μM) in HEPES buffer with 10% DMSO exhibited a gradual increase in the fluorescence intensity at 495 nm with increasing GSH concentration (Figure 19b). This response showcased the sensitivity of probe **18** to GSH, demonstrating a linear relationship and a low detection limit of 0.10 μM, indicating the probe's potential for precise GSH detection and differentiation from other thiols like Cys and Hcy. The probe facilitated targeted visualization of GSH within the lysosome of viable cells, offering an alternate approach for creating effective fluorescent probes for multi‐color bioimaging.^[87]^

**FIGURE 20:**
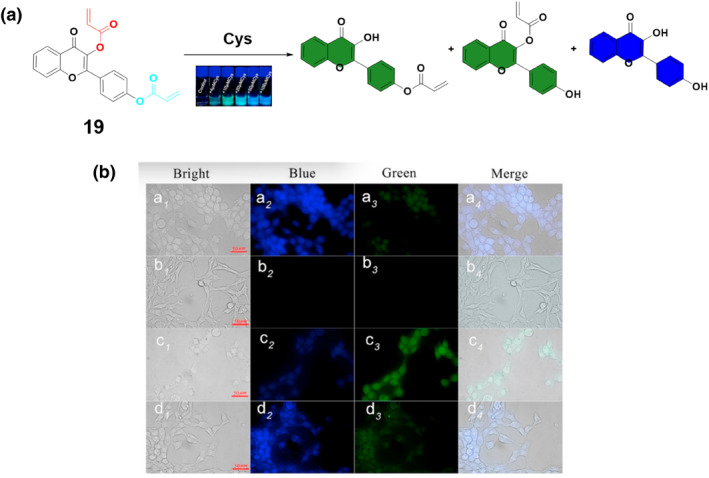
The design strategies of the dual‐site probe **19** (a). Fluorescence images of 4T1 cells showing probe **19** could be used for discriminating between different concentration ranges of Cys in living cells (b).

Dual acrylate sites, featuring two acrylate functional groups within a single molecule, offer a unique platform for targeted chemical reactions and sensing applications due to their versatile reactivity and specificity. In 2021, the Xu group developed a fluorescent probe **19** with dual acrylate sites to distinguish between various concentration ranges of Cys in living cells (Figure 20). The innovative design employed two identical reaction groups with varying steric hindrances to streamline the synthesis process and reduce interference from competing species. Probe **19** displayed varying reactivities towards Cys, resulting in identifiable fluorescence signals at both low and high concentration ranges. In the fluorescence images of 4T1 cells (Figure 20b), probe **19** demonstrated strong blue emission and weaker green emission in cells, indicating a reaction with endogenous Cys. Pre‐treatment with NEM to remove endogenous biothiols eliminated the fluorescence signal. Distinct emissions were observed with varying exogenous Cys concentrations, validating the probe's ability to differentiate between low and high Cys concentrations in living cells, as depicted by the fluorescence intensity and intensity ratio measurements. Probe **19** was effectively utilized to measure the concentration of Cys in living cells, offering a straightforward approach for designing dual‐site fluorescent probes to detect varying concentration levels of Cys, hence stimulating the development of future probe designs.^[88]^

**FIGURE 21:**
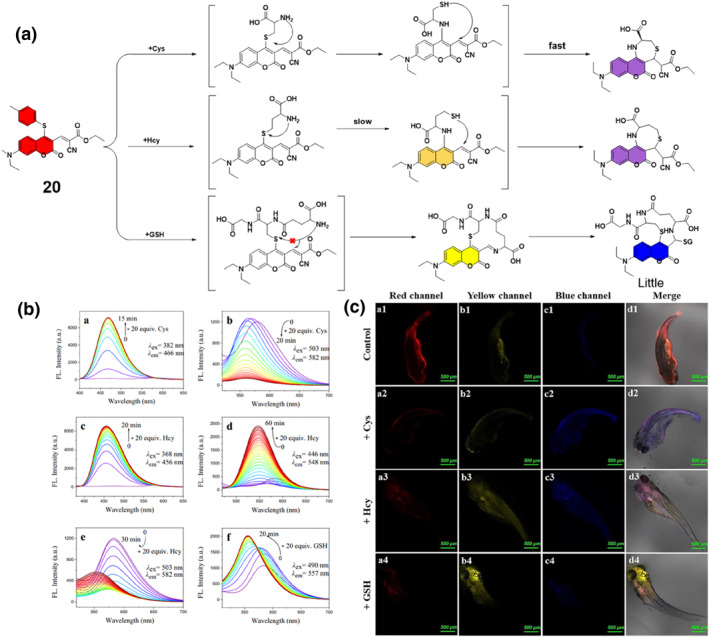
Response mechanisms of probe **20** with biothiols (a). Time‐dependent fluorescence spectra changes of probe **20** after treatment with thiols (b). Confocal images of zebrafish. (a1‐d1) Zebrafish treated with 15.0 μM probe **20**. (c) Zebrafish treated with biothiols for 30 min: (a2‐d2) for Cys (250 μM); (a3‐d3) for Hcy (250 μM); (a4‐d4) for GSH (5.0 mM), with distinguishable responses in red, blue, and yellow channels.

The substitution‐rearrangement‐cyclization technique, a versatile chemical strategy involving sequential substitution, rearrangement, and cyclization reactions, serves as a powerful tool in the synthesis of complex molecules. The Wang group employed a substitution‐rearrangement‐cyclization method to create a fluorescent probe **20** specifically developed for discerning and analyzing biothiols (Figure 21). Probe **20** utilized a dual‐site reaction with the analytes to control molecular conjugation. The sensing system employed a multi‐channel signal combination mode enabling the accurate detection of biothiols. The probe's efficacy was demonstrated by quantitatively detecting Cys, Hcy, and GSH in aqueous solutions. In Figure 21b, the time‐dependent fluorescence spectra changes of 10 μM probe **20** upon treatment with Cys, Hcy, and GSH revealed distinct fluorescent responses for each thiol. Cys induced a rapid quenching of orange‐red fluorescence and the appearance of strong blue emission. Hcy led to decreased fluorescence at 582 nm with new signals at 456 and 548 nm. GSH caused a blue shift to 557 nm and a change from orange to yellow emission, with excess GSH resulting in enhanced fluorescence at 475 nm. These responses enabled the discrimination of Cys, Hcy, and GSH based on their unique emission signal changes. In addition, probe **20** visualized the levels of biothiols in both living cells and zebrafish, demonstrating its sensitivity to biothiols.^[89]^

**FIGURE 22:**
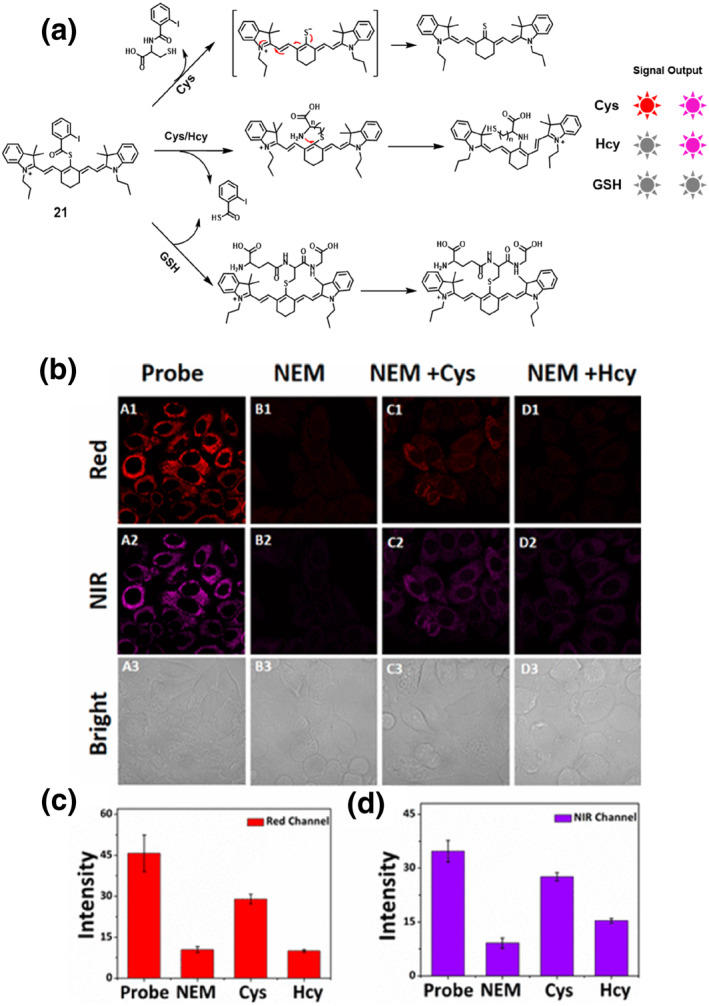
The proposed sensing mechanisms of probe **21** to distinguish Cys and Hcy (left). Fluorescence imaging of Cys and Hcy in HepG‐2 cells in red and NIR channels (a). Fluorescence intensity in the red channel (b) and in the NIR channel (c).

In 2022, the Guo group developed a fluorescent probe **21** that could differentiate between Cys and Hcy through single‐wavelength excitation with distinct dual emissions (Figure 22). Probe **21** was designed by combining a cyanine skeleton as a fluorophore with o‐iodobenzoate as the quenching group and leaving moiety via a thioester linker. The probe demonstrated a fluorescence response to Cys at wavelengths of 625 nm (red channel) and 740 nm (near‐infrared channel) while only displaying fluorescence turn‐on to Hcy at 740 nm (near‐infrared channel) and did not respond to GSH. The probe exhibited exceptional selectivity towards Cys and Hcy, accompanied by a rapid response. Simultaneous discriminative determination of Cys and Hcy were achieved using the built‐in self‐calibration of single excitation and dual emissions through the red and near‐infrared channels. The probe could detect exogenous Cys and Hcy in cells using two different emission channels with a single excitation. Furthermore, probe **21** could specifically target mitochondria and monitor changes in endogenous Cys levels within mitochondria using the red emission channel.^[90]^

**FIGURE 23:**
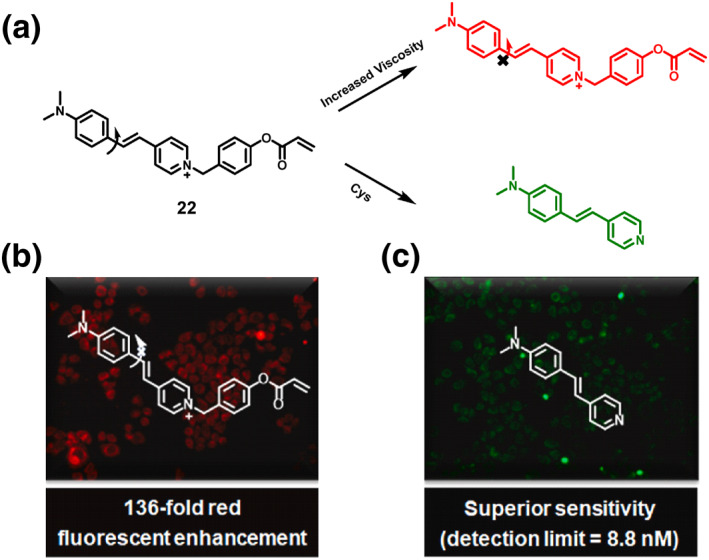
Rational design of a dual‐channel fluorescent probe **22** and its mechanism for simultaneously detecting Cys and viscosity.

In a significant leap forward in 2023, the Zheng group unveiled probe **22**, a pioneering dual‐channel fluorescent probe meticulously crafted to discern and differentiate biothiols, with a particular emphasis on Cys within biological systems (Figure 23). This innovative probe incorporated a 4‐p‐dimethylaminostyrylpyridinium unit, recognized for its viscosity‐sensing properties, and an acrylate unit specifically designed for Cys recognition. The probe's sensing method entailed the addition‐cyclization reaction between the probe and Cys, leading to the production of green fluorescence. Additionally, probe **22** displayed red fluorescence when the viscosity changed because the twisted intramolecular charge transfer process was suppressed. The group utilized fluorescence imaging to enable the simultaneous detection of Cys and viscosity in biological systems. They effectively showcased the probe's ability to visualize mitochondrial Cys and viscosity during drug‐induced hepatotoxicity, specifically in the case of acetaminophen‐induced hepatotoxicity, emphasizing the potential of the probe **22** for investigating abnormal physiological activities and the link between Cys and viscosity in important biological processes.^[91]^

## 3 DUAL MODE IMAGING TECHNIQUES FOR MOLECULAR PROBES IN LIVE SYSTEMS

Fluorescent probes have brought about a profound shift in how biothiols are detected and imaged in live systems, significantly advancing the field (Table 1). This capability provides comprehensive insight into the functions of biothiols in both physiological and pathological processes. The dual‐mode functionality typically includes a ratiometric response or a fluorescence turn‐on/off mechanism, enabling accurate measurement and imaging of biothiol levels in biological settings. This cutting‐edge imaging approach has significant potential for various applications, such as biomedical research, pharmaceutical development, and clinical diagnostics. Some research groups have developed fluorescent probes that incorporate smartphone imaging, fluorescent imaging, and/or multiphoton imaging to visualize and track biothiol levels in biosystems.[^93^, ^94^]

**TABLE 1:** Summary of fluorescent probes for dual recognition of biothiols.

No	Chemical structure	Target analyte	Limit of detection	Fluorescence response (nm)	Application	Ref
1	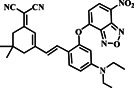	Hcy Cys/GSH	33.5 nM‐Hcy 33.2 nM/34.4 nM‐Cys/GSH	549/697‐ **λ**_**em**_ for Hcy 697‐ **λ**_**em**_ for Cys/GSH	HeLa cells	^[61]^
2	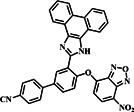	Cys Hcy/GSH	22.6 nM‐ Cys 31.2 nM‐Hcy 17.7 nM‐ GSH	470/550‐**λ**_**em**_ for Cys 470‐**λ**_**em**_ for Hcy/GSH	HeLa cells Zebrafish	^[62]^
3	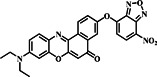	Cys GSH/H_2_S	0.05 μM ‐Cys 0.11 μM‐GSH 0.02 μM‐H_2_S	490/580‐**λ**_**abs**_ peaks for Cys 580 ‐**λ**_**abs**_ peak for GSH/H_2_S	HeLa cells, Zebrafish Mice	^[63]^
4	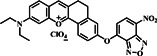	Cys/Hcy GSH	0.061 μM‐ Cys/Hcy 0.082 μM GSH	550/625 ‐**λ**_**em**_ for Cys/Hcy 625‐**λ**_**em**_ for GSH	HeLa cells, Zebrafish, *Arabidopsis thaliana*	^[64]^
5	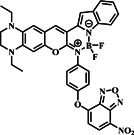	Cys/Hcy GSH	95.6 nM‐Cys 24.7 nM‐ Hcy 39.3 nM‐GSH	565/630‐ **λ**_**em**_ for Cys/Hcy 630‐ **λ**_**em**_ for GSH	HeLa cells, Zebrafish	^[65]^
6	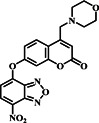	Cys/Hcy GSH	3.3 × 10^−8^ M‐ Cys 5.2 × 10^−8^ M‐ Hcy 3.9 × 10^−8^ M‐ GSH	400/470‐**λ**_**ex**_ Blue/Green fluorescence for Cys/Hcy Green fluorescence for GSH	HeLa cells	^[2]^
7	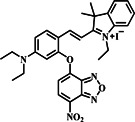	Hcy/Cys	*2.*81 μM*/2.*33 μM	543/592‐ **λ**_**em**_	MCF‐7 cells	^[66]^
8	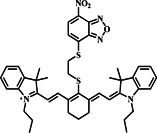	Cys/Hcy GSH	CY2 Probe 160 nM‐Hcy 94 nM‐Cys	550‐ Cys/Hcy 810‐ GSH	Living cells Mice	^[67]^
9	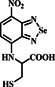	Cys/Hcy GSH	249.9 nM‐Cys 1.314 μM‐Hcy 19.5 nM‐GSH	600‐Cys/GSH 536‐ GSH	HepG2 cells	^[68]^
10	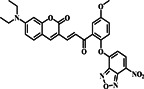	Cys/Hcy SO_2_	0.25 μM‐Cys 0.14 μM‐Hcy 0.05 μM‐SO_2_	540‐**λ**_**em**_ Cys/Hcy 590**‐λ**_**em**_ SO_2_	HepG2 cells Zebrafish	^[69]^
11	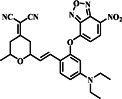	Cys/Hcy GSH	0.018 μM‐Cys 0.015 μM‐ Hcy 0.027 μM‐GSH	Green and Red channels‐Cys/Hcy Red fluorescence response‐GSH	HeLa cells	^[70]^
12	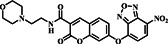	Hcy GSH Cys H_2_S	0.008 μM‐ Cys 0.030 μM‐Hcy 0.004 μM‐GSH 0.020 μM‐ H_2_S	465/565**‐λ**_**em**_ Cys/Hcy	HeLa cells Zebrafish	^[58]^
13	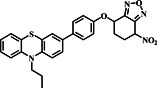	Cys/Hcy GSH	0.167 μM/0.172 μM Cys/Hcy	320/450‐(λ_ex/em_) GSH 450/540‐(λ_ex/em_) Cys/Hcy	HepG2 cells	^[92]^
14	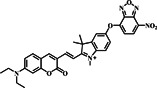	Cys/Hcy GSH SO_3_^2−^	0.21 μM‐ Cys 0.13 μM‐Hcy 0.14 μM‐GSH 3.06 μM‐SO_3_^2−^	Red, green and blue color changes	MCF‐7 cells Zebrafish MCF‐7 tumor model	^[78]^
15	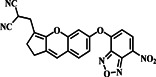	Cys/Hcy GSH	33.4 nM‐Hcy 33.5 nM‐Cys 33.5 nM‐ GSH	552/646 **λ**_**em**_ ‐ Cys/Hcy 646**‐λ**_**em**_ ‐ GSH	HepG2 cells Zebrafish	^[79]^
16	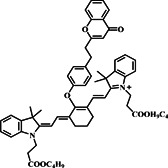	Cys Hcy GSH	24 nM‐GSH 32 nM‐Cys/Hcy	λ_ex_ −700 λ_em_ ‐ 810	U87 Glioma cells Mice	^[85]^
17	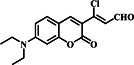	SO_2_ Cys	20 μM for SO_2_	λ_em_ for SO_2_ 500 and 565 nm	HeLa cells	^[86]^
18	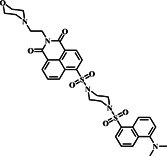	GSH	0.10 μM‐GSH	Green emission Orange emission	U‐87 MG cells	^[87]^
19	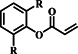	Cys	9.7 × 10^−8^ M	Green and blue emissions	4T1 cells	^[88]^
20	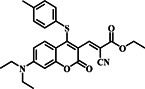	Cys Hcy GSH	0.10 μM‐Cys 0.28 μM‐Hcy 26.06 μM‐ GSH	Blue channel‐Cys Blue and yellow channels‐ Hcy Blue and yellow channels‐ GSH	HL‐7702 cells Zebra Fish	^[89]^
21	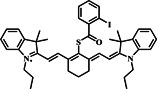	Cys Hcy GSH	0.55 μM‐Cys 0.35 μM‐Hcy	λ_em_‐625 and 740 nm ‐Cys λ_em_‐740 for Hcy	HepG‐2 cells	^[90]^
22	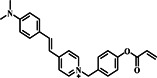	Cys	‐	Green‐Cys	HepG2 cells	^[91]^

**FIGURE 24:**
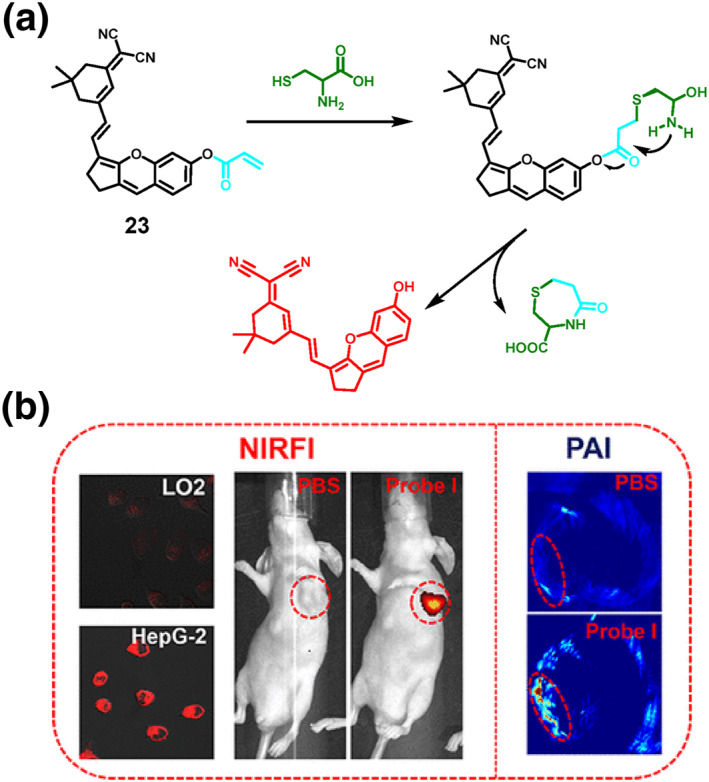
Schematic diagram of the structure and Cys detection mechanism (a) of the dual‐mode NIRF/PA probe **23** for imaging liver cancer in mice (b).

In 2022, the Chen group developed probe **23**, which combined Near Infrared fluorescence (NIRF) and Photoacoustic (PA) imaging modes (Figure 24). This probe allowed for highly sensitive and selective imaging of Cys in living organisms. Probe **23** was created using a versatile organic small‐molecule structure that incorporated dicyanoisophorone (DCI) as the fluorophore. It utilized the mechanism of donor‐π‐acceptor (D‐π‐A) system and employed an acryloyl group as a recognition site for Cys. When Cys was present, Probe **23** underwent an ICT mechanism, causing a notable enhancement in both NIRF and PA signals. The probe demonstrated exceptional selectivity and sensitivity with a detection limit of 10.6 nM for Cys. This enabled the detection of Cys in vitro, with a significant difference between the emitted wavelength and the Stokes shift. In addition, probe **23** facilitated in vivo imaging, specifically for monitoring liver cancer.^[95]^

**FIGURE 25:**
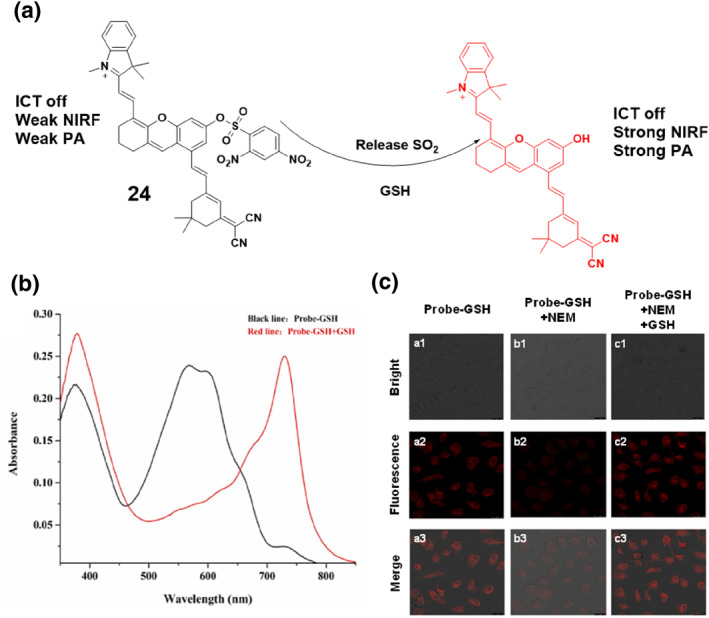
Illustration of probe **24**'s design and response to GSH (a). Spectral diagram of the reaction between probe **24** and GSH revealed distinct changes: three absorption bands initially observed at 600 nm, 585 nm, and 375 nm. Upon increasing the concentration of GSH, the absorption bands at 600 and 585 nm gradually diminished, while the band at 395 nm intensified. (b). Probe **24**'s reaction with GSH is visualized through cell imaging (c).

Around the same time in 2022, the Qin group developed probe **24** (Figure 25), which combined NIRF and PA imaging to visualize tumors in living organisms. The authors presented the idea of integrating NIR imaging and PA imaging, which provide non‐invasive and high‐resolution 3D reconstruction capabilities. Although PA imaging offers benefits in terms of tissue penetration depth and high ultrasonic spatial resolution,^[96]^ it is hindered by its low sensitivity and limited ability to detect fast biological processes. Dual‐modal imaging can overcome these limitations by combining the benefits of both modalities. Probe **24** employed a combination of xanthene‐based hemicyanines and dicyanoisophorone. This activation occurred through the overexpression of GSH in tumor microenvironments. Probe **24** demonstrated substantial improvements in both PA signal and NIRF intensity compared to currently available probes. The authors effectively utilized the probe to conduct in vivo imaging of mice with tumors using both NIRF and PA modalities.^[97]^

**FIGURE 26:**
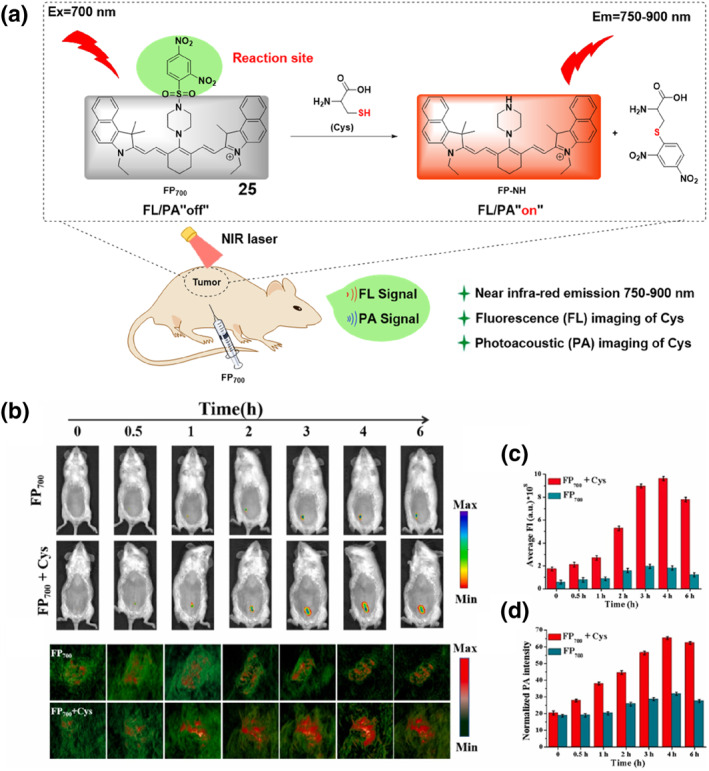
Rational designs of the probe **25** reporting Cys (top). The FL images of mice at different times before and after intraperitoneal injection of probe **25** and simultaneous injection of probe **25** and Cys (a). The histogram graphs of fluorescence intensity over time (λ_ex_ = 700 nm, λ_em_ = 790 nm) (b). The PA images were obtained by intraperitoneal injection of probe **25** and simultaneous injection of probe **25** and Cys at different times (c). The histogram of the PA signal over time (d).

The Zou group developed a cyanine‐based probe **25** that combined PA and fluorescence modalities (Figure 26). Probe **25** enabled real‐time imaging of endogenous Cys and in situ diagnosis of cervical cancer in living organisms. The probe design incorporated a cyanin dye that emits near‐infrared light as the fluorophore, linked to a recognition moiety for Cys, which consisted of a 2,4‐dinitrobenzenesulfonyl group. This design enabled the emission of fluorescence and photoacoustic signals upon stimulation at a wavelength of 700 nm. The sensing mechanism of probe **25** was dependent on the interaction between the recognition moiety and Cys, which led to enhanced signal response. This allowed for the non‐invasive and real‐time measurement of exogenous Cys levels within a living organism. The dual‐mode probe **25** combined the benefits of fluorescence imaging and PA for imaging. Fluorescence imaging exhibited superior sensitivity and precise selectivity, while PA demonstrated exceptional capability for imaging in living animals due to its ability to penetrate deep into tissues. The study determined that probe **25** effectively detected exogenous Cys in vitro by utilizing fluorescence and photoacoustic signals and precisely identified naturally occurring Cys in mice with tumors using a combination of imaging techniques.^[98]^

**FIGURE 27:**
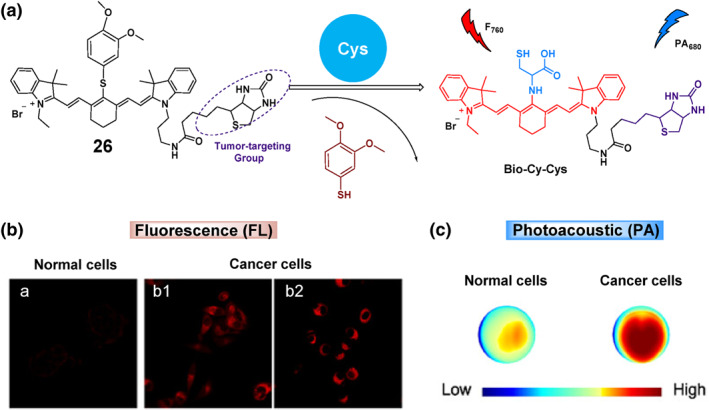
The chemical structure and biological application of probe **26** (top) to detect and track changes in Cys levels in normal cells and mice with tumors (bottom).

In 2023, the Gu group developed a novel tumor‐targeting probe named **26** for the dual‐modal imaging of Cys in living organisms (Figure 27). The probe employed a cyanine dye that emitted fluorescence in the NIR range and generated PA signals. It incorporated a recognition unit and a biotin structure for tumor targeting. Upon reacting with Cys, it triggered the activation of both NIR fluorescence and PA signals. Probe **26** was effectively utilized to detect Cys levels in living cells using two different methods and to track changes in Cys levels in mice with tumors. The study's key discovery was that **26** was the first probe with the ability to specifically target tumors and conduct dual‐modal imaging (using NIR fluorescence and PA) for cancer diagnosis.^[99]^

**FIGURE 28:**
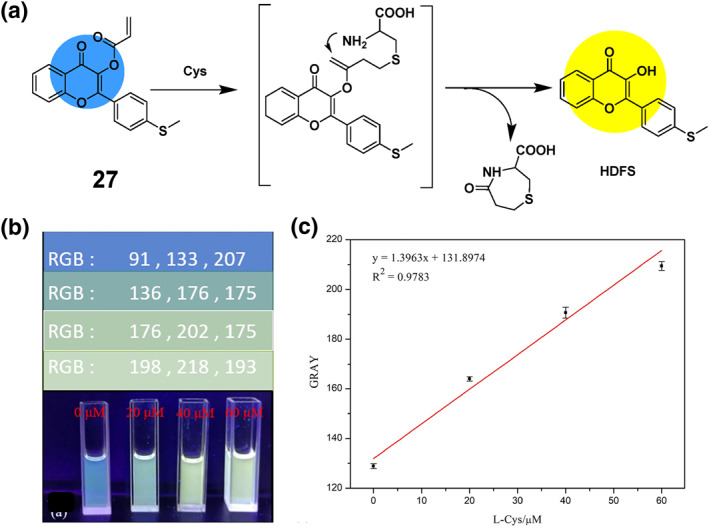
Detection mechanism of L‐Cys by probe **27** (top). Photographs of probe solutions with different probe **27** concentrations under 365 nm UV lamp (a). Fitting working curve of GRAY values versus probe **27** concentrations (b).

The implementation of imaging techniques for the quantitative detection of L‐Cys involves the use of a ratiometric probe **27** combined with smartphone imaging and fluorescent test strips (Figure 28). Smartphone imaging and fluorescent test strips offer several significant benefits for L‐Cys detection, including mobility, accessibility, user‐friendliness, rapidity, sensitivity, selectivity, and cost‐effectiveness.[^94^, ^100^, ^101^, ^102^, ^103^] For instance, in 2024, the Mei group developed a ratiometric flavonoid fluorescent probe **27**. The authors utilized smartphone imaging as a convenient and portable method for detecting L‐Cys, which demonstrated precise selectivity for L‐Cys. The test system solution saw a gradual color change from blue to yellow upon the addition of L‐Cys solution. The color variations of various L‐Cys concentrations were captured using a smart phone camera, as depicted in Figure 28a, while being exposed to 365 nm UV radiation. As demonstrated in Figure 28b, it was discovered that GRAY displayed a linear correlation with L‐Cys in the range of 0–60 μM, enabling the accurate detection of L‐Cys. The probe has other advantageous applications in cell imaging, smartphone fluorescence imaging, and fluorescence test strip detection.^[3]^

The application of two‐photon fluorescence microscopy demonstrates its utility as a powerful imaging tool for investigating biothiols in cellular environments, offering insights into their spatial distribution and temporal changes. Two‐photon fluorescence microscopy employs two photons with lower energy to stimulate fluorophores, advantageous over standard fluorescence microscopy with reduced photodamage, enhanced penetration depth, and greater spatial resolution. This imaging technique provides real‐time, subcellular level information about the localization and behavior of biothiols, enabling researchers to monitor changes in biothiol levels within living cells.[^104^, ^105^, ^106^, ^107^, ^108^]

**FIGURE 29:**
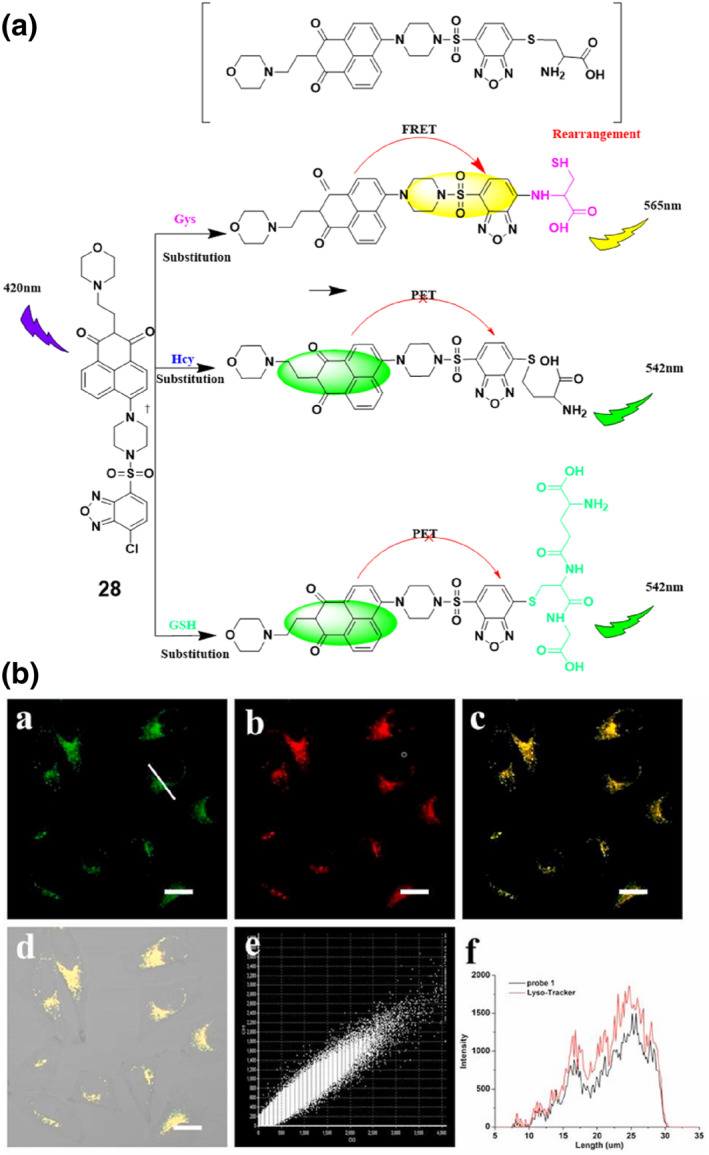
Detection mechanism of probe **28** on biothiols (a). Colocalization experiments in HeLa cells, (a) image of 5 μM probe **28** at 790 nm for 30 min, (b) image of 100 nM Lyso‐Tracker Red at 570 nm for 30 min, (c) The overlay image of (a) and (b). (d) The overlay image of bright‐field image and (c). (e) The fluorescence intensity correlation plot. (f) The intensity profile within the regions of interest across HeLa cells.

The Luo group constructed a two‐photon multi‐emissive fluorescent probe **28** for the discrimination of Cys and Hcy/GSH via an aromatic substitution‐rearrangement method (Figure 29).^[109]^ Probe **28** employed a sulfonyl benzoxadiazole (SBD) with a halogen chloride unit as the contact site. The probe allowed for excellent specificity, remarkable responsiveness, and minimal detection thresholds for biothiols. The probe facilitated the precise detection of thiol levels in human plasma and had the potential to be used for visualizing biothiols in lysosomes. Additionally, it enabled the live‐cell monitoring of GSH fluctuations using two‐photon fluorescence microscopy. The study emphasized the capability of the probe to efficiently monitor and comprehend the functions of biothiols in intricate physiological processes.

In summary, combining dual‐mode probes with advanced imaging techniques, such as photoacoustic imaging, fluorescent imaging, smartphone imaging, multi‐photon imaging, or other complex imaging techniques, allows for high‐resolution imaging in live systems. For instance, smartphone imaging and fluorescent test strips, offer portability for on‐the‐go testing and simplicity, providing user‐friendly and cost‐effective solutions for quick fluorescence‐based analysis. On the other hand, two‐photon fluorescence microscopy provides enhanced penetration depth and reduced photodamage, making it possible to image deeper tissue levels.

These methods enable the immediate tracking of biothiols in various biological settings, including cells, tissues, and entire organisms. By leveraging these advance imaging techniques, researchers can gain real‐time insights into the spatial distribution and temporal changes of thiols, enhancing our understanding of their roles in both physiological and pathological processes.

## 4 CONCLUSION AND OUTLOOK

Recent years have seen significant advancements in the development of fluorescent probes specifically designed for thiols, particularly with the introduction of probes with dual‐recognition capabilities. This review provided a concise overview of the design techniques, sensing mechanisms, biological applications, and imaging methods employed in molecular probes for thiols, specifically highlighting the progress made in the last 5 years. Despite significant advancements, future challenges remain. One important obstacle is the need for further enhancements in the sensitivity and selectivity of dual‐recognition probes. Accurate detection requires optimizing the interaction between the probe and target analytes while minimizing interference from biomarkers with similar structures or properties. Additionally, developing probes that are stable and functional in complex biological environments remains a critical challenge. To address these challenges, we propose several solutions that include enhancing sensitivity and selectivity through the exploration of new recognition motifs and the integration of signal amplification strategies. Secondly, there is a need to improve imaging methods and applications accomplished by developing subcellular targeting strategies and employing sophisticated imaging techniques. We believe that overcoming these challenges will contribute to this field and enable effective detection and monitoring of biothiols in biological systems.

## AUTHOR CONTRIBUTIONS

These authors made equal contributions to this paper.

## CONFLICT OF INTEREST STATEMENT

The authors declare that they have no known competing financial interests or personal relationships that could have appeared to influence the work reported in this paper.

## Data Availability

No data were used in the research described in the article.

## References

[1] X. Chen , Y. Zhou , X. Peng , J. Yoon , Chem. Soc. Rev. 2010, 39, 2120. 20502801 10.1039/b925092a

[2] X. Song , Y. Tu , R. Wang , S. Pu , Dyes Pigments 2020, 177, 108270.

[3] X. Mei , X. Yuan , Y. Yang , L. Liu , Y. Lin , L. Xie , X. Chai , K. Xu , G. Du , L. Zhang , J. Mol. Struct. 2024, 1312, 138486.

[4] C. Ge , F. Shen , Y. Yin , K. Chang , X. Zhang , P. Zhou , J. Li , Y. Liu , C. Lu , Talanta 2021, 223, 121758. 33298274 10.1016/j.talanta.2020.121758

[5] L. Pang , Y. Zhou , W. Gao , J. Zhang , H. Song , X. Wang , Y. Wang , X. Peng , Ind. Eng. Chem. Res. 2017, 56, 7650.

[6] Y. Yang , L. Zhang , X. Zhang , S. Liu , Y. Wang , L. Zhang , Z. Ma , H. You , L. Chen , Chem. Commun. 2021, 57, 5810. 10.1039/d1cc01110c 33999987

[7] X. Li , H. Ma , J. Qian , T. Cao , Z. Teng , K. Iqbal , W. Qin , H. Guo , Talanta 2019, 194, 717. 30609596 10.1016/j.talanta.2018.10.095

[8] X. Chen , Q. Wang , X. Zhuang , X. Chen , Q. Deng , C. Zhu , L. Lin , Int. J. Electrochem. Sci. 2024, 19, 100605.

[9] Y. Fukushima , S. Aikawa , Anal. Biochem. 2021, 621, 114185. 33826924 10.1016/j.ab.2021.114185

[10] J. Luchsinger , M.‐X. Tang , S. Shea , J. Miller , R. Green , R. Mayeux , Neurology 2004, 62, 1972. 15184599 10.1212/01.wnl.0000129504.60409.88

[11] M. P. Mattson , I. I. Kruman , W. Duan , Ageing Res. Rev. 2002, 1, 95. 12039451 10.1016/s0047-6374(01)00365-7

[12] H. Refsum , P. M. Ueland , O. Nygård , S. E. Vollset , Annu. Rev. Med. 1998, 49, 31. 9509248 10.1146/annurev.med.49.1.31

[13] H. Yuan , X. Li , J. Li , H. Zhang , M. Chen , Y. Wang , Chem. Phys. Lett. 2024, 843, 141245.

[14] X. Li , H. Wang , Y. Zhang , Q. Cao , Y. Chen , Chin. Chem. Lett. 2021, 32, 1541.

[15] H. J. Forman , H. Zhang , A. Rinna , Mol. Aspect. Med. 2009, 30, 1. 10.1016/j.mam.2008.08.006 PMC2696075 18796312

[16] S. K. Biswas , I. Rahman , Mol. Aspect. Med. 2009, 30, 60. 10.1016/j.mam.2008.07.001 PMC2699458 18760298

[17] D. M. Townsend , K. D. Tew , H. Tapiero , Biomed. Pharmacother. 2003, 57, 145. 12818476 10.1016/s0753-3322(03)00043-x PMC6522248

[18] A. P. Vellasco , R. Haddad , M. N. Eberlin , N. F. Hoehr , Analyst 2002, 127, 1050. 12195945 10.1039/b203832c

[19] Y. Ogasawara , Y. Mukai , T. Togawa , T. Suzuki , S. Tanabe , K. Ishii , J. Chromatogr. B Analyt Technol. Biomed. Life Sci. 2007, 845, 157. 10.1016/j.jchromb.2006.08.006 16962833

[20] R. Tang , C. Wang , X. Zhou , M. Feng , Z. Li , Y. Wang , G. Chen , Spectrochim. Acta Mol. Biomol. Spectrosc. 2023, 300, 122870. 10.1016/j.saa.2023.122870 37216722

[21] F. Dai , M. Zhao , F. Yang , T. Wang , C. Wang , Dyes Pigments 2020, 183, 108627.

[22] L. Yang , H. Xiong , Y. Su , H. Tian , X. Liu , X. Song , Chin. Chem. Lett. 2019, 30, 563.

[23] B. Dong , Y. Lu , N. Zhang , W. Song , W. Lin , Anal. Chem. 2019, 91, 5513. 31014068 10.1021/acs.analchem.9b01457

[24] W. Xie , J. Jiang , D. Shu , Y. Zhang , S. Yang , K. Zhang , Molecules 2023, 28, 4252. 37241992 10.3390/molecules28104252 PMC10222014

[25] J. Ma , X. Lu , Y. Guo , Z. Wang , Talanta 2023, 261, 124119. 36473742 10.1016/j.talanta.2022.124119

[26] J. Zhang , X. Ji , J. Zhou , Z. Chen , X. Dong , W. Zhao , Sensor. Actuator. B Chem. 2018, 257, 1076.

[27] S. Ding , G. Feng , Sensor. Actuator. B Chem. 2016, 235, 691.

[28] Y.‐G. Gao , Y. Zhang , Y.‐D. Shi , H.‐J. Hao , B. Gong , Z.‐L. Lu , Bioorg. Med. Chem. 2016, 24, 1550. 26924215 10.1016/j.bmc.2016.02.024

[29] M. Li , P. Cui , K. Li , J. Feng , M. Zou , X. Yu , Chin. Chem. Lett. 2018, 29, 992.

[30] H. Ge , Q. Ye , T. Zou , D. Zhang , H. Liu , R. Yang , TrAC, Trends Anal. Chem. 2024, 174, 117685.

[31] J. Guo , B. Fang , H. Bai , L. Wang , B. Peng , X.‐J. Qin , L. Fu , C. Yao , L. Li , W. Huang , TrAC, Trends Anal. Chem. 2022, 155, 116697.

[32] P. Wang , Y. Wang , N. Li , J. Huang , Q. Wang , Y. Gu , Sensor. Actuator. B Chem. 2017, 245, 297.

[33] F.‐F. Wang , Y.‐J. Liu , B.‐B. Wang , L.‐X. Gao , F.‐L. Jiang , Y. Liu , Dyes Pigments 2018, 152, 29.

[34] X. Dai , Z.‐Y. Wang , Z.‐F. Du , J. Cui , J.‐Y. Miao , B.‐X. Zhao , Anal. Chim. Acta 2015, 900, 103. 26572845 10.1016/j.aca.2015.10.023

[35] W. Fan , X. Huang , X. Shi , Z. Wang , Z. Lu , C. Fan , Q. Bo , Spectrochim. Acta Mol. Biomol. Spectrosc. 2017, 173, 918. 10.1016/j.saa.2016.10.060 27833065

[36] J. Zhang , F. Pan , Y. Jin , N. Wang , J. He , W. Zhang , W. Zhao , Dyes Pigments 2018, 155, 276.

[37] K. N. Bobba , G. Saranya , S. M. Alex , N. Velusamy , K. K. Maiti , S. Bhuniya , Sensor. Actuator. B Chem. 2018, 260, 165.

[38] J. Zhang , X. Ji , H. Ren , J. Zhou , Z. Chen , X. Dong , W. Zhao , Sensor. Actuator. B Chem. 2018, 260, 861.

[39] X.‐L. Liu , L.‐Y. Niu , Y.‐Z. Chen , Y. Yang , Q.‐Z. Yang , Biosens. Bioelectron. 2017, 90, 403. 27825881 10.1016/j.bios.2016.06.076

[40] H. Jiang , G. Yin , Y. Gan , T. Yu , Y. Zhang , H. Li , P. Yin , Chin. Chem. Lett. 2022, 33, 1609.

[41] L. Yu , S. Wang , K. Huang , Z. Liu , F. Gao , W. Zeng , Tetrahedron 2015, 71, 4679.

[42] Aruna , V. P. Verma , A. P. Singh , R. Shrivastava , J. Mol. Struct. 2024, 1295, 136549.

[43] W.‐T. Dou , H.‐H. Han , A. C. Sedgwick , G.‐B. Zhu , Y. Zang , X.‐R. Yang , J. Yoon , T. D. James , J. Li , X.‐P. He , Sci. Bull. 2022, 67, 853. 10.1016/j.scib.2022.01.014 36546238

[44] J.‐T. Hou , N. Kwon , S. Wang , B. Wang , X. He , J. Yoon , J. Shen , Coord. Chem. Rev. 2022, 450, 214232.

[45] Q. Yang , T. Lan , W. He , Dyes Pigments 2021, 186, 108997.

[46] H. Li , Y. Fang , J. Yan , X. Ren , C. Zheng , B. Wu , S. Wang , Z. Li , H. Hua , P. Wang , D. Li , TrAC, Trends Anal. Chem. 2021, 134, 116117.

[47] S. Lee , J. Li , X. Zhou , J. Yin , J. Yoon , Coord. Chem. Rev. 2018, 366, 29.

[48] R. Gui , H. Jin , X. Bu , Y. Fu , Z. Wang , Q. Liu , Coord. Chem. Rev. 2019, 383, 82.

[49] X. Tian , L. C. Murfin , L. Wu , S. E. Lewis , T. D. James , Chem. Sci. 2021, 12, 3406. 34163615 10.1039/d0sc06928k PMC8179477

[50] L. Liu , B. Liu , Y. Hao , J. Wang , X. Xu , X. Shang , J. Pharmaceut. Biomed. Anal. 2024, 239, 115876. 10.1016/j.jpba.2023.115876 38039872

[51] L. Wang , J. Wang , S. Xia , X. Wang , Y. Yu , H. Zhou , H. Liu , Talanta 2020, 219, 121296. 32887038 10.1016/j.talanta.2020.121296

[52] Z. Lu , X. Sun , M. Wang , H. Wang , C. Fan , W. Lin , Bioorg. Chem. 2020, 103, 104173. 32889381 10.1016/j.bioorg.2020.104173

[53] M. L. Circu , S. Stringer , C. A. Rhoads , M. P. Moyer , T. Y. Aw , Biochem. Pharmacol. 2009, 77, 76. 18840413 10.1016/j.bcp.2008.09.011 PMC2610527

[54] Z. Zhu , W. Liu , L. Cheng , Z. Li , Z. Xi , L. Yi , Tetrahedron Lett. 2015, 56, 3909.

[55] Y. A. Jeong , I. J. Chang , S.‐K. Chang , Sensor. Actuator. B Chem. 2016, 224, 73.

[56] S. Ding , W. Feng , G. Feng , Sensor. Actuator. B Chem. 2017, 238, 619.

[57] X. Ren , L. Liao , Z. Yang , H. Li , X. Li , Y. Wang , Y. Ye , X. Song , Chin. Chem. Lett. 2021, 32, 1061.

[58] X. Jing , F. Yu , W. Lin , Spectrochim. Acta Mol. Biomol. Spectrosc. 2020, 240, 118555. 10.1016/j.saa.2020.118555 32516703

[59] L. A. Montoya , M. D. Pluth , Anal. Chem. 2014, 86, 6032. 24852143 10.1021/ac501193r PMC4063329

[60] L. Yi , Z. Xi , Org. Biomol. Chem. 2017, 15, 3828. 28358164 10.1039/c7ob00332c

[61] S. Xu , J. Zhou , X. Dong , W. Zhao , Q. Zhu , Anal. Chim. Acta 2019, 1074, 123. 31159932 10.1016/j.aca.2019.05.008

[62] Z. Lu , Y. Lu , X. Sun , C. Fan , Z. Long , L. Gao , Bioorg. Chem. 2019, 92, 103215. 31541803 10.1016/j.bioorg.2019.103215

[63] L. Zhu , T. Zhang , Y. Ma , W. Lin , Sensor. Actuator. B Chem. 2020, 305, 127202.

[64] Y. Huang , Y. Zhang , F. g. Huo , Y. Liu , C. Yin , Sensor. Actuator. B Chem. 2019, 301, 127123.

[65] X. Ren , Y. Zhang , F. Zhang , H. Zhong , J. Wang , X. Liu , Z. Yang , X. Song , Anal. Chim. Acta 2020, 1097, 245. 31910966 10.1016/j.aca.2019.11.030

[66] K. Zhong , S. Zhou , X. Yan , S. Hou , X. Li , L. Tang , J. Lumin. 2020, 224, 117330.

[67] R. Li , H. Kassaye , Y. Pan , Y. Shen , W. Li , Y. Cheng , J. Guo , Y. Xu , H. Yin , Z. Yuan , Biomater. Sci. 2020, 8, 5994. 32990301 10.1039/d0bm01237h

[68] J. Yao , G. Yin , T. Yu , H. Li , P. Yin , Anal. Methods 2021, 13, 1358. 33635303 10.1039/d0ay02206c

[69] M. Zhang , Y. Zhang , F. Huo , J. Chao , S. Shuang , J. Photochem. Photobiol. Chem. 2022, 430, 113959.

[70] Y.‐Q. Hao , Y.‐T. Zhang , D.‐D. Zhu , L.‐J. Luo , L. Chen , Z.‐L. Tang , R.‐J. Zeng , M.‐T. Xu , S. Chen , Chin. J. Anal. Chem. 2022, 50, 100153.

[71] K. Surendran , S. P. Vitiello , D. A. Pearce , Pediatr. Nephrol. 2014, 29, 2253. 24217784 10.1007/s00467-013-2652-z PMC4018427

[72] D.‐D. He , W. Liu , R. Sun , C. Fan , Y.‐J. Xu , J.‐F. Ge , Anal. Chem. 2015, 87, 1499. 25569205 10.1021/ac5045912

[73] J.‐L. Zhu , Z. Xu , Y. Yang , L. Xu , Chem. Commun. 2019, 55, 6629. 10.1039/c9cc03299a 31119257

[74] U. T. Phan , B. Arunachalam , P. Cresswell , J. Biol. Chem. 2000, 275, 25907. 10852914 10.1074/jbc.M003459200

[75] D. Kand , T. Saha , M. Lahiri , P. Talukdar , Org. Biomol. Chem. 2015, 13, 8163, CCDC 980654 and 980655. For ESI and crystallographic data in CIF or other electronic format see DOI: 10.1039/c5ob00889a. 26140677 10.1039/c5ob00889a

[76] H. Zhang , L. Xu , W. Chen , J. Huang , C. Huang , J. Sheng , X. Song , ACS Sens. 2018, 3, 2513. 30465434 10.1021/acssensors.8b01101

[77] S. Tu , D. Li , T. Feng , Y. Le , L. Yan , L. Liu , J. Mol. Struct. 2024, 1295.

[78] J. Lan , L. Liu , Z. Li , R. Zeng , L. Chen , Y. He , H. Wei , Y. Ding , T. Zhang , Talanta 2024, 267, 125104. 37703779 10.1016/j.talanta.2023.125104

[79] K. Shen , Y. Hu , Q. Fei , E. Wang , J. Ren , G. Fan , F. Wang , J. Photochem. Photobiol. Chem. 2024, 448, 115341.

[80] Q. Liu , C. Sun , R. Dai , C. Yan , Y. Zhang , W.‐H. Zhu , Z. Guo , Coord. Chem. Rev. 2024, 503, 215652.

[81] J. Duan , X. Ouyang , Z. Jiang , Z. Liu , X. Wang , Spectrochim. Acta Mol. Biomol. Spectrosc. 2024, 316, 124330. 10.1016/j.saa.2024.124330 38685160

[82] J. Zhang , W. Wang , J. Shao , J. Chen , X. Dong , Coord. Chem. Rev. 2024, 516, 215986.

[83] L. Zhou , L. Xie , C. Liu , Y. Xiao , Chin. Chem. Lett. 2019, 30, 1799.

[84] Y. Wu , H.‐H. Han , L. He , L. Li , Y. Zang , J. Li , X.‐P. He , Y. Ding , W. Cao , T. D. James , Chem. Commun. 2023, 59, 5051. 10.1039/d2cc06425a 37021645

[85] Y. Xu , R. Li , X. Zhou , W. Li , U. Ernest , H. Wan , L. Li , H. Chen , Z. Yuan , Talanta 2019, 205, 120125. 31450407 10.1016/j.talanta.2019.120125

[86] Q. Zhang , Z. Cui , Q. Wang , G. Zheng , Sensor. Actuator. B Chem. 2019, 295, 79.

[87] Z. Xu , M.‐X. Zhang , G. Li , X. Chen , S. H. Liu , H. Chen , J. Yin , Dyes Pigments 2019, 171, 107685.

[88] Z. Xu , S. Si , Z. Zhang , H. Tan , T. Qin , Z. Wang , D. Wang , L. Wang , B. Liu , Anal. Chim. Acta 2021, 1176, 338763. 34399901 10.1016/j.aca.2021.338763

[89] Y. Wang , Y. Yue , F. Huo , K. Ma , C. Yin , Spectrochim. Acta Mol. Biomol. Spectrosc. 2021, 261, 120026. 10.1016/j.saa.2021.120026 34091363

[90] T. Guo , X. Chen , W. Qu , B. Yang , R. Tian , Z. Geng , Z. Wang , Anal. Chem. 2022, 94, 5006. 35294170 10.1021/acs.analchem.1c04895

[91] Y.‐L. Zheng , R. Yu , M. Li , C. Fan , L. Liu , H. Zhang , W. Kang , R. Shi , C. Li , Y. Li , J. Wang , X. Zheng , Heliyon 2023, 9, e22276. 38053901 10.1016/j.heliyon.2023.e22276 PMC10694328

[92] S. Tu , D. Li , T. Feng , Y. Le , L. Yan , L. Liu , J. Mol. Struct. 2024, 1295, 136583.

[93] D. Quesada‐González , A. Merkoçi , Biosens. Bioelectron. 2017, 92, 549. 27836593 10.1016/j.bios.2016.10.062

[94] M. Zhang , X. Cui , N. Li , Mater. Today Bio 2022, 14, 100254. 10.1016/j.mtbio.2022.100254 PMC9034388 35469257

[95] Z. Chen , B. Wang , Y. Liang , L. Shi , X. Cen , L. Zheng , E. Liang , L. Huang , K. Cheng , Anal. Chem. 2022, 94, 10737. 35876030 10.1021/acs.analchem.2c01372

[96] Y. Liu , L. Nie , X. Chen , Trends Biotechnol. 2016, 34, 420. 26924233 10.1016/j.tibtech.2016.02.001 PMC5600199

[97] J. Qin , H. Tian , F. Kong , Y. Guo , W. Du , C. Zhang , H. Gu , Y. Li , Sensor. Actuator. B Chem. 2022, 371, 132522.

[98] X. Zou , Y. Zhao , W. Lin , Anal. Chim. Acta 2023, 1239, 340713. 36628718 10.1016/j.aca.2022.340713

[99] Q.‐S. Gu , Z.‐C. Yang , J.‐J. Chao , L. Li , G.‐J. Mao , F. Xu , C.‐Y. Li , Anal. Chem. 2023, 95, 12478. 37555783 10.1021/acs.analchem.3c02134

[100] D. Zhang , F. Zhang , S. Wang , S. Hu , Y. Liao , F. Wang , H. Liu , Spectrochim. Acta Mol. Biomol. Spectrosc. 2023, 290, 122285. 10.1016/j.saa.2022.122285 36592594

[101] S. Wang , J. Xu , F. Yue , L. Zhang , N. Bi , J. Gou , Y. Li , Y. Huang , T. Zhao , L. Jia , Food Chem. 2024, 451, 139410. 38670024 10.1016/j.foodchem.2024.139410

[102] B. Purohit , A. Kumar , K. Mahato , P. Chandra , Curr. Opin. Biomed. Eng. 2020, 13, 42.

[103] A. Roda , M. M. Calabretta , D. Calabria , C. Caliceti , L. Cevenini , A. Lopreside , M. Zangheri , in Comprehensive Analytical Chemistry (Eds: I. Palchetti , P.‐D. Hansen , D. Barceló , Eds.), Vol. 77, Elsevier 2017, pp. 237–.Chapter Eight ‐ Smartphone‐Based Biosensors for Bioanalytics: A Critical Review.

[104] Z.‐H. Zhang , C.‐C. Li , J. Qu , H. Zhang , K. Liu , J.‐Y. Wang , Spectrochim. Acta Mol. Biomol. Spectrosc. 2022, 278, 121361. 10.1016/j.saa.2022.121361 35569200

[105] H. W. Lee , V. Juvekar , D. J. Lee , H. M. Kim , TrAC, Trends Anal. Chem. 2023, 165, 117128.

[106] Y. Ma , Y. Zhao , L. Xia , J. Huang , Y. Gu , P. Wang , Anal. Chim. Acta 2018, 1035, 161. 30224135 10.1016/j.aca.2018.06.036

[107] M. J. Green , H. Ge , S. E. Flower , C. Pourzand , S. W. Botchway , H.‐C. Wang , N. Kuganathan , G. Kociok‐Köhn , M. Li , S. Xu , T. D. James , S. I. Pascu , RSC Chem. Biol. 2023, 4, 1082. 38033726 10.1039/d3cb00112a PMC10685793

[108] V. Juvekar , H. W. Lee , D. J. Lee , H. M. Kim , TrAC, Trends Anal. Chem. 2022, 157, 116787.

[109] W. Luo , S. Zhang , Q. Meng , J. Zhou , R. Jin , X. Long , Y.‐P. Tang , H. Guo , Talanta 2021, 224, 121833. 33379051 10.1016/j.talanta.2020.121833

